# ﻿In Memoriam: Cytogeneticist Dr. Sc. Ninel A. Petrova (1940–2024) — life and scientific heritage

**DOI:** 10.3897/compcytogen.18.138747

**Published:** 2024-12-18

**Authors:** Valentina Kuznetsova, Paraskeva Michailova, Andrey Przhiboro, Natalia Khabazova

**Affiliations:** 1 Department of Karyosystematics, Zoological Institute, Russian Academy of Sciences, Universitetskaya emb. 1, 199034 St. Petersburg, Russia; 2 Research Group of Cytotaxonomy and Evolution, Institute of Biodiversity and Ecosystem Research, Bulgarian Academy of Sciences, Tsar Osvoboditel blvd. 1, 1000 Sofia, Bulgaria; 3 Laboratory of Freshwater and Experimental Hydrobiology, Zoological Institute, Russian Academy of Sciences, Universitetskaya emb. 1, 199034 St. Petersburg, Russia

**Keywords:** Bibliography, biography, Chironomidae, chromosomal rearrangements, Diptera, evolution, polytene chromosomes, research highlights, Simuliidae, systematics

## Abstract

The article is dedicated to the memory of Dr. Sc. Ninel A. Petrova, who passed away on 18 April 2024, and to her scientific heritage. Ninel was born on 28 March 1940 in Leningrad, the former Soviet Union. From 1959 to 1964, she studied at the Faculty of Biology and Soil Science of the Leningrad State University. After graduating from the university, Ninel joined the Zoological Institute of the Russian Academy of Sciences, where she worked for almost 60 years, rising from a research assistant to a leading researcher. Her scientific research focused on the structure and evolution of polytene chromosomes of two families of Diptera, black flies (Simuliidae) and non-biting midges (Chironomidae). Ninel has published nearly 200 scientific articles, three monographs and one monograph chapter and has become one of the leading experts in her field. Along with scientific activities, Ninel worked for 25 years as the Scientific Secretary of the Specialized Scientific Council for the Defense of Dissertations at the Zoological Institute.

## ﻿Introduction

**Figure 1.**
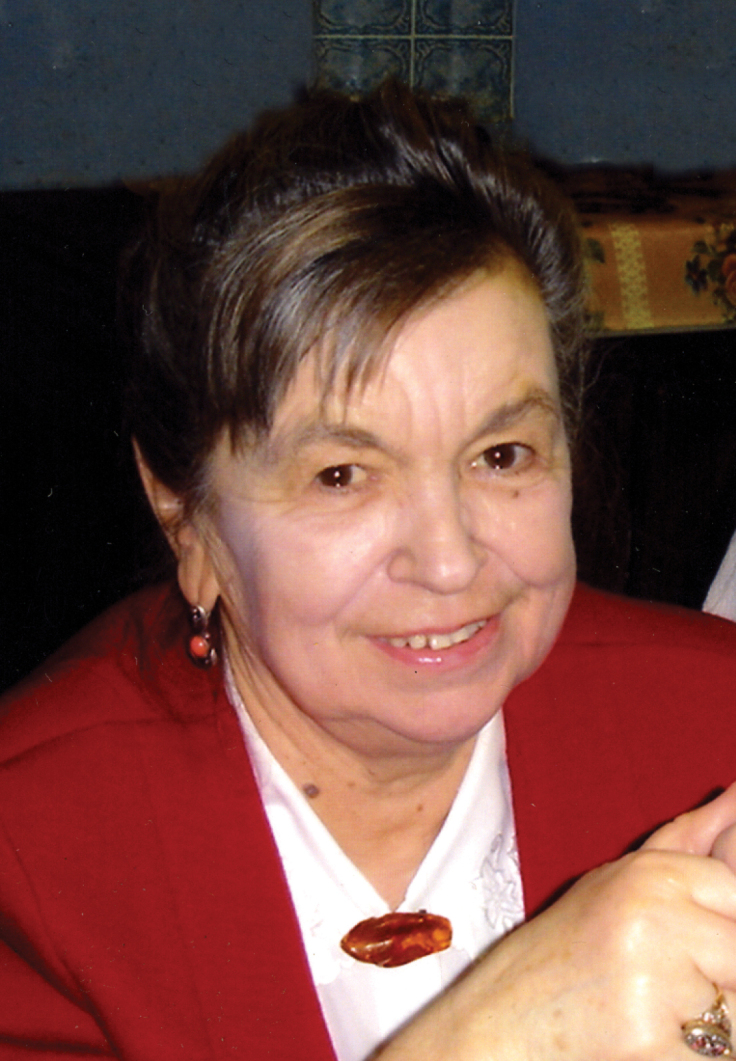
Dr. Ninel A. Petrova (photo by P. Michailova).

**Figure 2.**
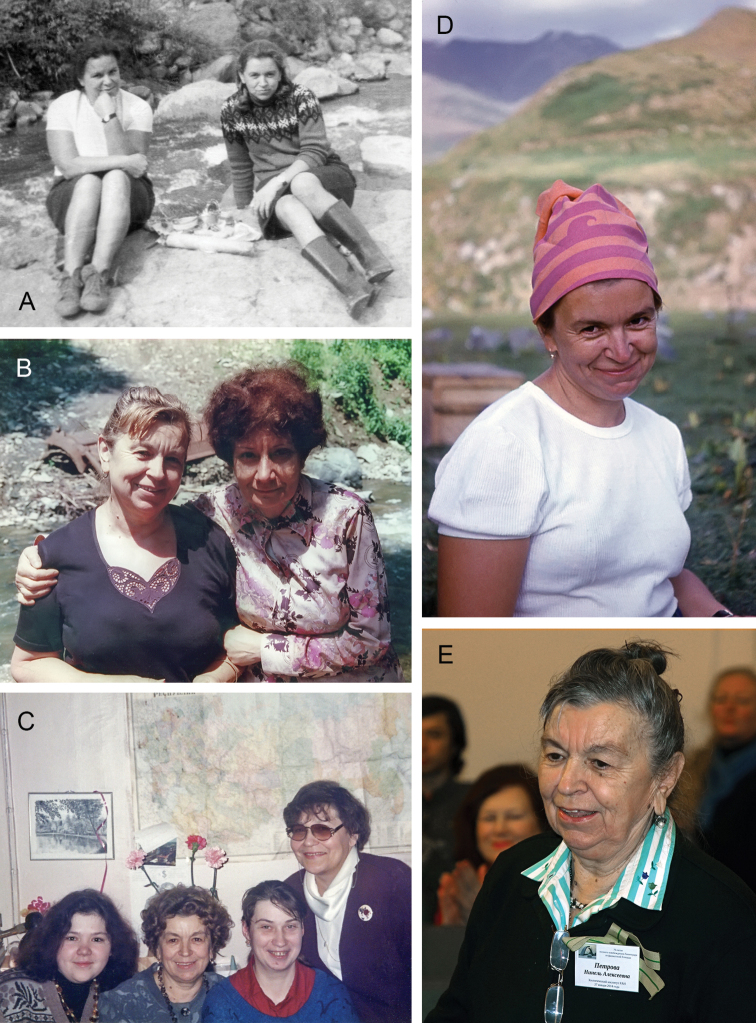
Photos of Ninel A. Petrova **A** with L.A. Chubareva (at left), expedition to Azerbaijan, 1969 **B** with E.A. Kachvoryan (at right), 1990s (?) **C** with N.V. Golub (at left), N.V. Khabazova and V.G. Kuznetsova (at right), at Zoological Institute (St. Petersburg), September 1997 **D** expedition to Tajikistan (?), 1975 **E** at Zoological Institute (St. Petersburg), 27 January 2014. (**A–D** photos from personal archive of N.A. Petrova; **E** photo by A.A. Przhiboro).

**Figure 3.**
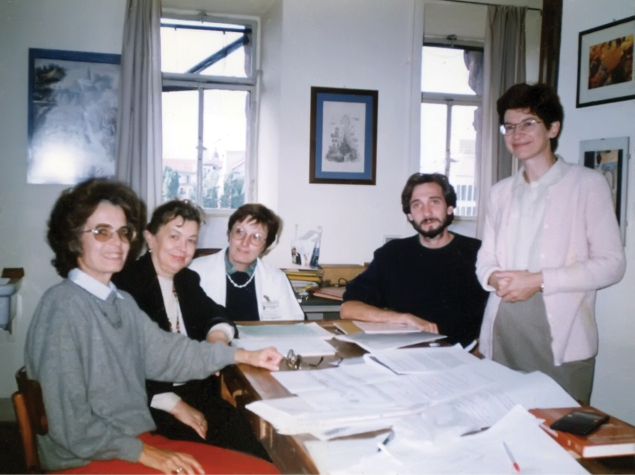
Photo of Ninel A. Petrova with Bulgarian and Italian colleagues, participants of the NATO project (Turin, 2010). P. Michailova (at left), L. Ramella, S. Bovero and G. Sella (at right).

**Figure 4.**
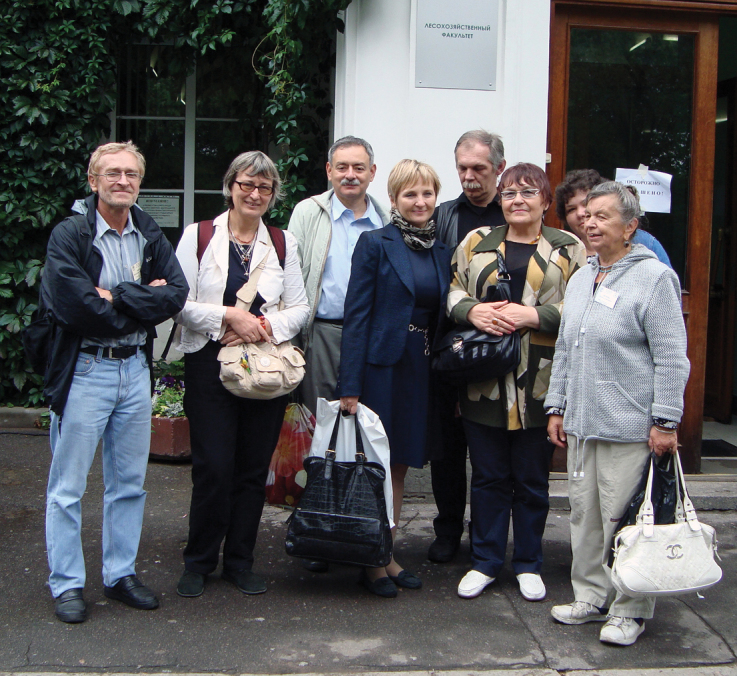
Photo of Ninel A. Petrova with colleagues, participants of the XIV All-Russian Entomological Congress, Section of karyosystematics (2012, St. Petersburg). N.A. Petrova, N.V. Golub, V.G. Kuznetsova, S.V. Zhirov, N.V. Durnova, V.E. Gokhman, S.M. Grozeva, and V.A. Lukhtanov (right to left).

Dr. Sc. Ninel A. Petrova (Figs 1, 2A–E, 3, 4) passed away on 18 April 2024 after nearly 60 years of work devoted to the cytogenetic and cytotaxonomic studies of insects of the dipteran families Chironomidae (non-biting midges) and Simuliidae (black flies). Her contributions to the understanding of the structure and evolution of polytene chromosomes of Diptera cannot be overestimated. There can be very few scientists working on the comparative cytogenetics and karyosystematics of these groups who have not cited the publications of Ninel Petrova.

## ﻿Life and times

Ninel Petrova was born in the town of Petrodvorets near Leningrad (now Peterhof in St. Petersburg) on 28 March 1940. She never saw her father, who died in the 1939–1940 Soviet-Finnish War. Ninel was just over a year old when the Great Patriotic War (1941–1945) began, and she and her mother were evacuated from besieged Leningrad to the Ryazan Province, to the town of Pronsk. Life in evacuation, hungry and unsettled, was a traumatic experience. Ninel’s memories of this period, based largely on her mother’s accounts, can be found in the book “War’s Tragic Memory: The Great Patriotic War in the Memoirs of the Staff of the Zoological Institute of the Russian Academy of Sciences” (2021).

After the war, the family returned to Leningrad. Ninel’s mother remarried, and Ninel had a stepfather. According to her stories, he was a very kind man and did everything possible for her upbringing. In 1957, Ninel graduated from school. She was interested in biology and, after working for two years in different places, she entered the Leningrad State University (now St. Petersburg State University), the Faculty of Biology and Soil Science where she specialized in plant genetics, specifically rye, at the Department of Genetics and Breeding. Her scientific supervisor was Prof. Vasily S. Fedorov (1903–1976), a famous geneticist and breeder, a remarkable scientist, the creator of the genetic collection of rye and the first domestic variety of tetraploid rye “Leningradskaya Tetra”. During the dark period of ‘Lysenkoism’ in the former Soviet Union, Prof. Fedorov introduced students to the basics of genetics in lectures formally devoted to the criticism of classical genetics. He was a wonderful supervisor who played an important role in the shaping of Ninel Petrova as a scientist and whom she remembered with warmth and gratitude all her life. After graduating and defending her diploma project, Ninel joined the Zoological Institute of the Russian Academy of Sciences in Leningrad as a research assistant in the newly established Laboratory of Karyosystematics and Population Genetics and remained there for the whole of her working career. Ninel was very lucky with her teachers. The head of this laboratory was Prof. Lidia A. Chubareva, one of the founders of the cytotaxonomic approach to insect research in the former Soviet Union. During her years of work at the Institute, Ninel rose from a laboratory assistant to a leading researcher. She not only participated in the scientific life of the laboratory, but also for 25 years held the position of the Scientific Secretary of the Specialized Scientific Council for the Defense of Candidate (Ph. D.) and Doctoral (Dr. Sc.) dissertations at the Zoological Institute in the specialties of “Entomology” and “Parasitology”. In this position, she contributed to the formation of the scientific team and the maintenance of the scientific community of the institute. Many colleagues who defended their dissertations in those years were very grateful for the help and advice she gave them, especially regarding dissertation documentation and preparation for defense.

## ﻿Research highlights

Dr. Sc. Ninel Petrova was a scientist who made great and very valuable contributions to the cytotaxonomy and cytogenetics of the large insect order Diptera. Her research focused on two large and practically important families, Simuliidae (black flies) and Chironomidae (non-biting midges), and on their giant polytene chromosomes. These chromosomes are found in the interphase nuclei of the salivary glands of larvae and consist of thousands of DNA strands resulting from multiple replication cycles without separation of sister chromatids. As Ninel has shown in numerous publications, rearrangements detected in polytene chromosomes can be successfully used to solve the problems of systematics and phylogenetics of these insects at various taxonomic levels, from the separation of closely related species to the analysis of phylogenetic relationships between higher taxa.

Under the supervision of Prof. Lidia Chubareva, who was one of the world leaders in cytotaxonomic studies of black flies, Ninel worked on comparative cytogenetics of this highly important group of insects. These studies resulted in a series of publications, first jointly with her supervisor (e.g., Chubareva and Petrova 1968, 1969) and then independently (Petrova 1972, and others) or with other colleagues (see List of main publications at the end of the article and Supplementary file 1). Many of her taxonomic publications have successfully combined karyological and morphological approaches. On the example of several taxa of black flies (the genera *Cnephia* Enderlein, 1921, *Helodon* Enderlein, 1921, *Schoenbaueria* Enderlein, 1921, *Austrosimulium* Tonnoir, 1925, and others), Ninel Petrova demonstrated how karyological characteristics can be used to clarify the status and position of taxa as well as the phylogenetic relationships between them. In one of her earlier publications (Petrova et al. 1971), devoted to a large and widespread genus *Simulium* Latreille, 1802 and published in co-authorship with Prof. Ivan A. Rubtsov, a well-known Russian expert in the systematics of Simuliidae, the prospect of using chromosomal characters to distinguish closely related species differing only slightly in external morphology was clearly demonstrated. In 1975, Ninel successfully defended her Candidate (Ph. D.) dissertation “Comparative karyological study of bloodsucking black flies of the genera *Cnephia* End., *Metacnephia* Crossk. and *Sulcicnephia* Rubz. (Diptera, Simuliidae)”.

After nearly a decade devoted to the study of Simuliidae, in the late 1970s Ninel began to apply karyotaxonomic methods and approaches to another large and diverse dipteran family, Chironomidae, without leaving her first object, Simuliidae. In 1992, she successfully defended her Doctoral dissertation entitled “Polytene chromosomes of chironomids and simuliids, their use in the study of the systematics and evolution of these insect groups (Diptera: Chironomidae, Simuliidae)” and was awarded a well-deserved scientific degree “Doctor of Sciences” (D. Sc.). Ninel has made a great contribution to the knowledge of structural and functional organization of dipteran polytene chromosomes and to the cytotaxonomy of Simuliidae and Chironomidae. Ongoing research on the former led to the publication (in co-authorship with Prof. Chubareva) of the world’s first review of karyotypes of black flies (Chubareva and Petrova 2008). This book (with the subtitle ‘Atlas’) contains the detailed cytophotomaps of polytene chromosomes of 124 species in 32 genera of black flies of Russia and neighboring countries (descriptions and drawings of taxonomically significant morphological characters of the studied species were also provided). This monograph has become a desktop book for all specialists dealing with comparative cytogenetics of black flies.

Since then, Ninel has focused her efforts mostly on the study of polytene chromosomes and karyosystematics of the family Chironomidae (although she has also published important papers on black flies during these years), which is of both theoretical and practical interest. Chironomid larvae are widely distributed and abundant in aquatic ecosystems, where they play an important role. Ninel carefully studied the morphological characteristics of polytene chromosomes of species from different phylogenetic lineages and subfamilies within the family Chironomidae. Her original chromosome maps of chironomid species from five subfamilies are not only of great taxonomic importance, but have also been successfully used to trace species relatedness at the cytogenetic level (Petrova et al. 2000).

Ninel studied the structural and functional organization of polytene chromosomes of chironomids originating from different regions of Russia and from other countries, including Mongolia, Lithuania, Italy, Bulgaria, Belarus, Ukraine and others (Petrova 1990; Petrova et al. 1986, 1992, 2000, 2004, 2013, 2014a, b). During her life, she participated in many scientific expeditions to collect the material, e.g. to the European North, the Caucasus, Tajikistan and Kyrgyzstan (Fig. 2A, B, D).

Ninel’s studies on the variability of polytene chromosomes are also very important because they take into account both seasonal and geographical variability in their band structure (Petrova 1990; Petrova et al. 2004, 2014a, b). She has made a remarkable contribution to the use of polytene chromosomes of model chironomid species to trace metal pollution in various aquatic ecosystems. Because of her authority as a brilliant expert on chironomid cytogenetics, she was invited to participate in two NATO projects named, respectively, “Polytene chromosomes as a model for heavy metal-induced genome instability” (2004–2006) and “Pollution of water resources assessed by genome alterations in midges (2008–2010)”. As part of these projects carried out jointly with Bulgarian and Italian colleagues (Fig. 3), Ninel studied the influence of heavy metals on the structural and functional organization of chromosomes of model species of chironomids collected in Russia, Bulgaria and Italy. She has been actively involved in the development of these projects and has established the presence of both multiple heritable and a number of somatic chromosomal rearrangements (Michailova et al. 1996, 1998, 2012; Petrova and Michailova 2002; Petrova 2013; Michailova and Petrova 2015). During these years, Ninel conducted classes and lectures for students from Turin and Milan. Using her knowledge and skills, the research team conducted a number of laboratory experiments with heavy metal ions, which have led to practically important conclusions about the genotoxic effect of some of them (Michailova et al. 2001a, b). This study confirmed that the chironomid genome is a very sensitive structure and responds much more strongly to toxicants than the external morphology of larvae.

The extraordinary self-sacrifice of N. Petrova in the name of science deserves special mention. She conducted herself in the field like a true scientist, collecting the scientific material. Without a moment’s hesitation, she went to Chernobyl (Ukraine) after the accident at the nuclear power station (26 April 1986) and collected chironomids to study the influence of radiation on the structural and functional organization of their polytene chromosomes. This activity resulted in her research on the chromosomal variability of chironomid species from Chernobyl (Michailova and Petrova 1994; Petrova and Michailova 1996a, b).

During her long life in science, Ninel published about 200 scientific articles, three monographs (Chubareva and Petrova 2008; Petrova 2013; Petrova and Zhirov 2022) and a chapter (Belyanina et al. 1983) in an important collective monograph. Using combined analysis of chromosomes and morphological characters, she discovered and described five new species and two genera of Simuliidae (Petrova 1977, 1983; Chubareva and Petrova 1981; Petrova et al. 1995; see the list of taxa below). Also, as part of an international research team, she described a new chironomid species *Polypedilumpembai* Cornette et al. from Malawi, southeastern Africa (Cornette et al. 2017). This species is notable for the ability of its larvae to survive the dry season in a completely dehydrated ametabolic state known as anhydrobiosis, similar to that in its widely known congener, *P.vanderplanki* Hinton, 1951. Although *P.pembai* has been described based on a suite of features including adult, larval and pupal morphology and DNA sequencing results, its independence was initially inferred based on the chromosomal data obtained by Ninel (Petrova et al. 2015). One species of Simuliidae and one genus of Chironomidae were named in honor of Ninel Petrova (see below).

An important result of Dr. Petrova’s research on chironomids was her last monograph “Structure of polytene chromosomes and morphology of chironomid larvae (Diptera, Chironomidae)” (Petrova and Zhirov 2022). In this book, Ninel together with her former Ph. D. student, the late Sergey V. Zhirov (Kuznetsova et al. 2019), reviewed the data on the polytene chromosomes in larvae belonging to five subfamilies of Chironomidae. This book is mostly based on the authors’ original materials and contains original microphotographs of polytene chromosomes. In addition to the chromosomal data, it includes the data on larval morphology. This monograph summarized valuable comparative information on polytene chromosome morphology and chromosomal polymorphism in different populations and species of Chironomidae.

Ninel was a researcher who collaborated productively with colleagues in Russia and some other countries such as Bulgaria, Italy and Armenia. She enjoyed passing on her knowledge and skills to younger colleagues who came to her laboratory and teaching them working with polytene chromosomes. The Zoological Institute and the Department of Karyosystematics, where she worked, have lost a remarkable scientist devoted to science in general.

## ﻿Scientific presentations

Ninel Petrova has repeatedly made excellent presentations at national (Soviet and All-Russian) and international conferences and congresses. We will list just a few of them, such as all five International Conferences on Karyosystematics of Invertebrate Animals (in 1979 and 2006 in Leningrad/St. Petersburg, in 1991 in Cheboksary, in 1997 in Moscow, in 2010 in Novosibirsk and in 2016 in Saratov), International Symposia on Chironomidae (e.g., in 1997 in Freiburg, Germany), Congresses of the All-Russian Entomological Society (e.g., in 2002 and 2012 (Fig. 4) in St. Petersburg, in 2007 in Krasnodar, and in 2017 in Novosibirsk), Congresses of the Russian Society of Geneticists and Breeders (e.g., in 2014 in Rostov-on-Don and in 2019 in St. Petersburg), All-Russian Dipterological Symposia (e.g., in 2016 in Krasnodar and in 2020 in Voronezh), the X International Balbiani Ring Workshop (in 2021 in Varna, Bulgaria) and many others. Her reports have always been received with great interest.

## ﻿The taxa described by Ninel Petrova

### ﻿In Chironomidae

*Polypedilumpembai* Cornette, N. Yamamoto, M. Yamamoto, Kobayashi, Petrova, Gusev, Shimura, Kikawada, Pemba et Okuda, 2017

### ﻿In Simuliidae

*Levitinia* Chubareva et Petrova, 1981

*Levitiniatacobi* Chubareva et Petrova, 1981

*Metacnephiapamiriensis* Petrova, 1977

*Metacnephiaparaskevae* Petrova, Chubareva et Kachvoryan, 1995

*Prosimuliumpamiricum* Chubareva et Petrova, 1983

*Rubzovia* Petrova, 1983 [described as a genus; currently recognized as a subgenus of *Simulium*]

*Rubzoviavantshi* Petrova, 1983 [current name: Simulium (Rubzovia) vantshi (Petrova, 1983)]

## ﻿The taxa named in honor of Ninel Petrova

### ﻿In Chironomidae

*Ninelia* E. Makarchenko et M. Makarchenko, 2004 [described with the wording: ‘described in honor of … N.A. Petrova, a kind and sympathetic person who devoted most of her life to chironomid karyology, moral (and not only moral) support of young (and not so young) Russian chironomidologists’].

### ﻿In Simuliidae

*Sulcicnephiapetrovae* Rubtsov, 1976 [described with the wording: ‘named after Ninel Alekseevna Petrova, who collected the material in difficult conditions of the high-mountain country’].

Note: All listed taxonomic names are currently valid.

## ﻿Awards

Ninel Petrova was repeatedly awarded prizes and diplomas for her scientific achievements. One of the most significant awards for her was the medal “For the Rescue of the Perished”, which she received in the framework of the Decree of the President of the Russian Federation № 419 of 21.04.2006 “On awarding state awards of the Russian Federation to active participants of liquidation of consequences of the accident at the Chernobyl Nuclear Power Plant”.

## ﻿Personal characteristics

Ninel was a sweet, friendly and very creative person. Her interactions with colleagues and friends were always very kind and even-keeled. She was passionate about music, classical Russian literature and painting, and enjoyed attending concerts and exhibitions in her beautiful city of St. Petersburg. Ninel passed on her hobbies to her daughter Olga, who teaches literature and Russian language at a school in St. Petersburg.

## ﻿Conclusions

Ninel passed away at the age of 84 years old. Throughout her life she carried a sincere interest in science, working in the same laboratory for almost 60 years and becoming one of the leading specialists in her field. Ninel’s colleagues and friends will always remember her as a wonderful professional and a very kind person. We express our deepest grief on the death of our dear friend and colleague. She will always live in our hearts and memories.

## ﻿List of main publications by Ninel Petrova

The publications mentioned in the above text are asterisked. A more complete list of publications by N.A. Petrova with comments (compiled by A. Przhiboro) is given as Suppl. material 1.

### ﻿1968

*Chubareva LA, Petrova NA (1968) Homologous lines of chromosome polymorphism in the natural populations of blackflies (Diptera, Simuliidae). Tsitologiya 10(10): 1248–1256. [In Russian with English summary].

### ﻿1969

*Chubareva LA, Petrova NA (1969) Karyological peculiarities of *Helodonferrugineus* Wahlb. in relation to some questions of systematics. Tsitologiya 11(2): 234–241. [In Russian with English summary].

### ﻿1971

Chubareva LA, Petrova NA, Petrukhina TE (1971) The structural peculiarities of the polytene chromosomes and the taxonomy of the black flies (Diptera, Simuliidae). Tsitologiya 13(6): 784–789. [In Russian with English summary].

*Petrova NA, Rubtsov IA, Chubareva LA (1971) On the position of *Simulium* (*Schönbaueria*) *gigantea* Rubz. in the system of classification of simuliids (Morphological and karyological characters in the systematics). Parazitologiya 5(1): 40–50. [In Russian with English translation in Parasitology, 1972, 1(1): 45–57].

### ﻿1972

*Petrova NA (1972) Karyological features of the Karelian black flies [sic!] of the genus *Cnephia* End. Tsitologiya 14(6): 769–773, 2 plates. [In Russian with English summary].

### ﻿1973

Petrova NA (1973) A comparative karyological analysis of three genera of the family Simuliidae (Diptera). Tsitologiya 15(8): 1055–1059. [In Russian with English summary].

Petrova NA (1973) A comparative karyological study of 3 species of black-flies of the genus *Metacnephia* Crosskey (Diptera, Simuliidae) from Armenia. Tsitologiya 15(4): 439–445, 3 plates. [In Russian with English summary].

### ﻿1974

Petrova NA (1974) Inversional polymorphism in natural populations of two species of black flies (Diptera, Simuliidae). Genetika 10(1): 80–89. [In Russian with English summary].

### ﻿1975

Chubareva LA, Petrova NA (1975) Karyotype of the plesiomorphic New Zealand species *Austrosimuliumtillyardi* and its genetical relations with some other genera of the family Simuliidae (Diptera). Zoologicheskii Zhurnal 54(4): 552–558. [In Russian with English summary].

*Petrova NA (1975) Comparative karyological study of blood-sucking black flies of the genera *Cnephia* End., *Metacnephia* Crossk. and *Sulcicnephia* Rubz. (Diptera, Simuliidae). Candidate of Biological Sciences Dissertation, Leningrad: Zoological Institute, Academy of Sciences of the USSR, 242 pp. [In Russian].

### ﻿1976

Chubareva LA, Rubtsov IA, Petrova NA (1976) Morphological and karyological similarities and differences in Palearctic and Neotropical species of the genus *Hemicnetha* End. (Diptera, Simuliidae). Entomologicheskoe Obozrenie 55(2): 452–457. [In Russian with English translation in Entomological Review, 1976, 55(2): 137–142].

### ﻿1977

*Petrova NA (1977) A new species of black flies; *Metacnephiapamiriensis* sp. n. (Simuliidae) from Pamir. Parazitologiya 11(3): 210–212, 1 plate. [In Russian with English summary].

Petrova NA, Chubareva LA, Kuzmenko KN (1977) The karyotypes of five species of Chironomidae (Diptera). Tsitologiya 19(8): 900–905. [In Russian with English summary].

Rubtsov IA, Petrova NA (1977) Blackflies of the tribe Cnephiini (Diptera, Simuliidae) and diagnoses of the genera *Cnephia* Enderlein and *Astega* Enderlein. Entomologicheskoe Obozrenie 56(3): 691–697. [In Russian with English translation in Entomological Review, 1977, 56(3): 145–149].

### ﻿1978

Maksimova FL, Petrova NA (1978) Geographical variability of karyotype in *Chironomusplumosus* (Diptera, Chironomidae). Zoologicheskii Zhurnal 57(12): 1816–1826. [In Russian with English summary].

Petrova NA, Chubareva LA (1978) The peculiarities of the karyotype of *Prodiamesaolivacea* Meig. (Diptera, Chironomidae, Orthocladiinae). Tsitologiya 20(10): 1208–1211. [In Russian with English summary].

Petrova NA, Maksimova FL (1978) The role of chromosome rearrangements in the speciation of chironomids (Diptera, Chironomidae). Genetika 14(7): 1201–1207. [In Russian with English translation in Soviet Genetics, 1978, 14: 849–853].

### ﻿1979

Chubareva LA, Petrova NA (1979) Main characters of karyotypes of blackflies (Diptera, Simuliidae) of the world. In: Chubareva LA (Ed.) Karyosystematics of the Invertebrate Animals. Leningrad: Zoological Institute, Academy of Sciences of the USSR, pp. 58–95. [In Russian with English summary].

Petrova NA (1979) Chromosomal rearrangements distinguish species of the genus *Metacnephia* (Diptera, Simuliidae). In: Nartshuk EP (Ed.) Ecological and Morphological Principles of Diptera Systematics (Insecta). Materials of Symposium (13–15 September 1978, Voronezh), Leningrad: Zoological Institute AN SSSR, pp. 79–81. [In Russian with English translation in Skarlato OA (Ed.), 1979, Systematics of Diptera (Insecta): Ecological and Morphological Principles. New Delhi, India: Amerind Publishing Co. Pvt. Ltd., pp. 125–128].

### ﻿1980

Chubareva LA, Petrova NA (1980) A method for making of cytological preparations for caryological study of Diptera. In: Chubareva LA (Ed.) New Data on Karyosystematics of Dipterous Insects. Trudy Zoologicheskogo Instituta AN SSSR, 95: 73–80. [In Russian with English summary; signed for press on 29 December 1980 and possibly therefore actually published in 1981]

Maksimova FL, Petrova NA (1980) On the problem of sympatric species evolution in chironomids (Diptera, Chironomidae). In: Lukina EV (Ed.) Problems of Population Cytogenetics of Plants and Animals, pp. 70–76. [In Russian].

Pankratova VYa, Chubareva LA, Petrova NA (1980) On the systematics of some *Chironomus* species (Chironomidae) from the Lake Sevan. In: Chubareva LA (Ed.) New Data on Karyosystematics of Dipterous Insects. Trudy Zoologicheskogo Instituta AN SSSR, 95: 50–54. [In Russian with English summary].

Petrova NA (1980) Cytological sex determination of blackflies (Simuliidae). In: Chubareva LA (Ed.) New Data on Karyosystematics of Dipterous Insects. Trudy Zoologicheskogo Instituta AN SSSR, 95: 55–58. [In Russian with English summary].

Petrova NA (1980) Karyological research of Orthocladiinae (Diptera, Chironomidae). Genetica 52–53: 275–279. https://doi.org/10.1007/BF00121837

### ﻿1981

*Chubareva LA, Petrova NA (1981) A new genus of black flies (Diptera, Simuliidae) from Tadzhikistan. Entomologicheskoe Obozrenie 60(4): 898–900. [In Russian with English translation in Entomological Review, 1982, 60(4): 140–144].

Petrova NA (1981) Morphological peculiarities of polytene chromosomes in two black fly species (Diptera, Simuliidae). Tsitologiya 23(11): 1317–1320. [In Russian with English summary].

Petrova NA, Chubareva LA, Zolotaryova LV, Kaliberdo TA (1981) The karyotypes of chironomids from the Bratsk Reservoir (Diptera). Tsitologiya 23(10): 1180–1187. [In Russian with English summary].

### ﻿1982

Chubareva LA, Petrova NA (1982) Cytogenetic method of chromosome analysis in chironomids. In: Akhrorov F (Ed.) Methodical Guide to Chironomid Research. Dushanbe: Donish, pp. 64–73. [In Russian].

Petrova NA, Zolotareva LV (1982) Morphology and karyotype of a larva of *Micropsectra* sp. (Diptera, Chironomidae) from the East Pamir. Zoologicheskii Zhurnal 61(10): 1605–1607. [In Russian with English summary].

### ﻿1983

*Belyanina SI, Maksimova FL, Bukhteeva NM, Il’inskaya NB, Petrova NA, Chubareva LA (1983) Chapter 2. Systematics and morphology. Division 2. Karyotype. In: Sokolova NJu (Ed.) *Chironomusplumosus* L. (Diptera, Chironomidae). Systematics, Morphology, Ecology, Production. Moscow: Nauka Publishers, pp. 61–96. [In Russian].

Chubareva LA, Petrova NA (1983) A new species of blackflies of genus *Prosimulium* (Roub.) [sic!] (Simuliidae, Diptera) from Pamir. In: Nartshuk EP (Ed.) Diptera (Insecta), Their Systematics, Geographic Distribution and Ecology (15–17 September 1982, Belaya Tserkov’). Leningrad: Zoological Institute AN SSSR, pp. 141–144. [In Russian].

*Petrova NA (1983) A new genus and species of buffalo gnats (Diptera, Simuliidae) from West Pamir. Zoologicheskii Zhurnal 62(12): 1911–1915. [In Russian with English summary].

Petrova N (1983) The karyotype and unstable associations of polytene chromosomes in *Syndiamesanivosa* (Diptera, Chironomidae). Zoologicheskii Zhurnal 62(1): 69–74. [In Russian with English summary].

Petrova NA (1983) Population variability of blood-sucking black fly *Sulcicnephiaovtchinnikovi* (Simuliidae). Parazitologiya 17(6): 470–473. [In Russian with English summary].

### ﻿1984

Chubareva LA, Petrova NA (1984) B chromosomes of black flies (Simuliidae, Diptera). Genetika 20(4): 570–578. [In Russian with English translation in Soviet Genetics, 1984, 20(4): 446–453].

Michailova P, Petrova N (1984) Initial stage of sympatric divergency in species of the genus *Glyptotendipes* Kieff. (Diptera, Chironomidae). Caryologia 37(4): 293–307. https://doi.org/10.1080/00087114.1984.10797708

Petrova NA, Chubareva LA (1984) A list of species of the black flies (Diptera, Simuliidae) of Tajikistan. In: Nartshuk EP, Zlobin VV (Eds) Diptera (Insecta) of the Fauna of the USSR and Their Significance in Ecosystems (15–17 September 1982, Belaya Tserkov’). Leningrad: Zoological Institute AN SSSR, pp. 99–106. [In Russian with English translation in Entomological Review, 1992, 71(3): 39–46].

### ﻿1985

Il’inskaya NB, Petrova NA (1985) B-chromosomes of *Chironomusplumosus* (Diptera, Chironomidae). Genetika 21(10): 1671–1679. [In Russian with English summary].

Petrova NA, Feher LV (1985) Chromosomal polymorphism in *Glyptotendipesparipes* (Edw.) (Diptera, Chironomidae). Tsitologiya 27(6): 710–713. [In Russian with English summary].

### ﻿1986

Petrova NA (1986) The value of karyological characters for the taxonomy, systematics and evolution of chironomids. In: Kolesnikov NN, Istomina AG (Eds) Evolution, Speciation and Systematics of Chironomids. Novosibirsk: Institute of Cytology and Genetics of the Academy of Sciences of the USSR, pp. 29–35. [In Russian].

*Petrova N, Kiknadze I, Michailova P (1986) Integration of species in the plumosus-group of Chironomidae. In: Aukshtikal’nene AM, Permyakova LV (Eds) A System of Species Integration. Vilnius, pp. 138–161. [In Russian with English summary].

Petrova NA, Michailova P (1986) The population karyological studies of some Chironomidae species (Diptera, Chironomidae). Tsitologiya 28(7): 727–734. [In Russian with English summary].

### ﻿1987

Michailova P, Petrova NA (1987) Peculiarities of the karyotype of Micropsectragr.notescens (Diptera, Chironomidae) from different populations. Tsitologiya 29(9): 1056–1060. [In Russian with English summary].

Petrova NA (1987) Chromosome numbers in the Chironomidae. In: Nartshuk EP (Ed.) Diptera and Their Importance for Animal Husbandry and Agriculture (17–19 September 1986, Alma-Ata). Leningrad: Zoological Institute AN SSSR, pp. 136–143. [In Russian].

### ﻿1988

Il’inskaya NB, Petrova NA, Dyomin SYu (1988) Seasonal variations of chromosomal polymorphism in *Chironomusplumosus* L. (Diptera, Chironomidae). Genetika 24(8): 1393–1401. [In Russian with English translation in Soviet Genetics 24: 967–974].

Makarchenko EA, Petrova NA (1988) Chironomids of the subfamily Tanypodinae of the Far East of the USSR. I. Morpho-karyological description of *Macropelopiaparanebulosa* Fittkau. In: Levanidova IM, Makarchenko EA (Eds) Fauna, Systematics and Biology of Freshwater Invertebrates. Vladivostok, pp. 28–35. [In Russian].

### ﻿1989

Balushkina EA, Petrova NA (1989) Functioning of populations of chironomids in hypersalt lakes of the Crimea. In: The Investigations of the Water Ecosystem [sic!]. Trudy Zoologicheskogo Instituta AN SSSR (Proceedings of the Zoological Institute of the Academy of Sciences of the USSR), 205: 129–139. [In Russian].

Michailova P, Petrova NA (1989) Microevolution differentiation of Pseudodiamesagr.branickii Nowicki (Diptera, Chironomidae). Tsitologiya 31(7): 824–828. [In Russian with English summary].

Petrova NA (1989) Characteristics of the karyotypes of midges (Diptera, Chironomidae) of the world fauna. I. Subfamilies Telmatogetoninae, Podonominae, Tanypodinae, Diamesinae, Prodiamesinae and Orthocladiinae. Entomologicheskoe Obozrenie 68(1): 107–120. [In Russian with English translation in Entomological Review, 1989, 68(4): 68–85].

Petrova NA (1989) Chironomids of the subfamily Tanypodinae. II. Chromosome numbers of three species of Tanypodinae (Diptera, Chironomidae). In: Drjanovska OA (Ed.) Fourth National Conference on Cytogenetics with International Participation, 2–6 October, 1989, Vratsa, Bulgaria, pp. 192–194. [In Russian].

Petrova NA (1989) Results and prospects of the karyological study of chironomids. In: Dévai G (Ed.) Advances in Chironomidology: Proceedings of the Xth International Symposium on Chironomidae, Debrecen, 25–28 July, 1988. Pt. 1. Systematics, Molecular Biology, Cytology, Population Genetics, Zoogeography and Phenology. (Acta Biologica Debrecina. Supplementum Oecologica Hungarica, 2). Debrecen, pp. 295–304.

Petrova NA, Michailova PV (1989) Chromosome polymorphism of natural populations of *Endochironomusalbipennis* Meig. (Diptera, Chironomidae). Tsitologiya 31(10): 1200–1205. [In Russian with English summary].

### ﻿1990

*Petrova N (1990) Characteristics of chironomid karyotypes (Diptera, Chironomidae) of the world fauna II. Subfamily Chironominae. Entomologicheskoe Obozrenie 69(1): 193–214. [In Russian].

Petrova NA, Il’yinskaya NB (1990) Revision of populations of chironomids of plumosus group based on the analysis of fixed and fluctuating inversions. In: Khmeleva NN (Ed.) Species in Area: Biology, Ecology and Productivity of Water Invertebrates. Minsk: Navuka i Tekhnika, pp. 69–74. [In Russian].

### ﻿1991

Michailova P, Petrova N (1991) Chromosome polymorphism in geographically isolated populations of *Chironomusplumosus* L. (Chironomidae, Diptera). Cytobios 67: 161–175.

Petrova NA (1991) Chromosomal rearrangements in three species of chironomids (Diptera, Chironomidae) from the Chernobyl zone. Genetika 27(5): 836–848. [In Russian with English summary].

### ﻿1992

Il’inskaya NB, Petrova NA (1992) One more time about the standard karyotype of *Chironomusplumosus* L. and cytodiagnostics of its sibling species (Diptera, Chironomidae). Zoologicheskii Zhurnal 71(12): 76–86. [In Russian with English translation as “On the typical karyotype of *Chironomusplumosus* and cytodiagnostic of “plumosus–group” species (Diptera, Chironomidae)” in Entomological Review, 1993, 72(4): 135–147].

Kachvoryan EA, Chubareva LA, Petrova NA (1992) [as “1991”] Comparative karyological analysis of two species of blackflies of the genus *Tetisimulium* Rubz. (Simuliidae, Diptera). In: Richter VA, Zlobin VV (Eds) Advantages of Entomology in USSR. Diptera: Systematics, Ecology, Medical and Veterinary Importance. St. Petersburg: Zoological Institute, Russian Academy of Sciences, pp. 145–148. [In Russian].

Petrova NA (1992) [as “1991”] Chromosomal aberrations in natural populations of chironomids from water bodies of Chernobyl. In: Richter VA, Zlobin VV (Eds) Advantages of Entomology in USSR. Diptera: Systematics, Ecology, Medical and Veterinary Importance. St. Petersburg: Zoological Institute, Russian Academy of Sciences, pp. 12–15. [In Russian].

*Petrova N (1992) Polytene chromosomes of chironomids and simuliids and their role for studying the systematics and evolution of these groups. Doctor of Biological Sciences Dissertation. St. Petersburg: Zoological Institute, Russian Academy of Sciences, 411 pp. and 154 pp. (Supplement). [In Russian].

Il’inskaya NB, Petrova NA (1992) One more time about the standard karyotype of *Chironomusplumosus* L. and cytodiagnostics of its sibling species (Diptera, Chironomidae). Zoologicheskii Zhurnal 71(12): 76–86. [In Russian with English translation as: On the typical karyotype of *Chironomusplumosus* and cytodiagnostic of “plumosus–group” species (Diptera, Chironomidae), in Entomological Review, 1993, 72(4): 135–147.]

*Petrova NA, Michailova PV, Maximova FL, Il’inskaya NB (1992) The standard karyotype of *Chironomusplumosus* L. (Diptera, Chironomidae). Cytobios 70: 185–189.

Zelentsov NA, Petrova NA, Erbaeva EA (1992) Karyotype and morphology of *Acricotopuslucens* Zett. (Diptera, Chironomidae) from Mongolia. Entomologicheskoe Obozrenie 71(2): 295–301. [In Russian with English translation in Entomological Review, 1993, 72(3): 28–34].

### ﻿1994

*Michailova P, Petrova N (1994) Cytogenetic characteristics of *Chironomusbalatonicus* Devai, Wülker, Scholl (Diptera, Chironomidae) from the Chernobyl region. Cytobios 79: 15–29.

Petrova NA, Ivanchenko OV, Kerkis IE (1994) Cytogenetic structure of natural populations of *Chironomusbalatonicus*. Tsitologiya 36(5): 469–478. [In Russian with English summary].

### ﻿1995

*Petrova NA, Chubareva LA, Kachvoryan EA (1995) A new species of black flies, *Metacnephiaparaskevae* sp. n. (Diptera, Simuliidae), from the East Pamir (Tajikistan). Entomologicheskoe Obozrenie 74(4): 899–901. [In Russian with English translation in Entomological Review, 1996, 75(9): 227–231].

Shobanov NA, Petrova NA (1995) Karyotype peculiarities in *Chironomussaxatilis* Wülker et al., 1981 (Diptera, Chironomidae) from the Siberian Arctic region and a possible origin of neocentromeres [in chromosome AE (pseudothummi cytocomplex)]. Tsitologiya 37(7): 586–592. [In Russian with English summary].

### ﻿1996

Chubareva LA, Petrova NA, Kachvoryan EA (1996) Morpho-karyotypical features of four species of blackflies (Diptera: Simuliidae). Parazitologiya 30(1): 3–12. [In Russian with English summary].

Il’inskaya NB, Petrova NA (1996) Regularities of appearance of inversion polymorphism in the centre and at borders of range of *Chironomusplumosus*. In: Shobanov NA, Zinchenko TD (Eds) Ecology, Evolution and Systematics of Chironomidae. Togliatti, Borok, pp. 8–17. [In Russian].

Kachvoryan EA, Chubareva LA, Petrova NA, Mirumyan LS (1996) Frequency changes of B chromosomes in synanthropic species of bloodsucking blackflies (Diptera, Simuliidae). Genetika 32(5): 637–640. [In Russian with English translation in Russian Journal of Genetics, 1996, 32(5): 554–557].

Kerkis IE, Gordadze PR, Petrova NA, Chubareva LA (1996) One more time about the karyotype of *Prodiamesaolivacea* (Prodiamesinae, Chironomidae, Diptera). Tsitologiya, 38(3): 384–389. [In Russian with English summary].

*Michailova P, Petrova N, Ramella L, Sella G, Todorova J, Zelano V (1996) Cytogenetic characteristics of a population of *Chironomusriparius* Meigen, 1804 (Diptera, Chironomidae) from a polluted Po river station. Genetica 98: 161–178. https://doi.org/10.1007/BF00121364

Petrova NA, Il’inskaya NB, Kaidanov LZ (1996) Adaptiveness of inversion polymorphism in Chironomusplumosus (Diptera, Chironomidae): spatial distribution of inversions over species range. Genetika 32(12): 1629–1642. [In Russian with English translation in Russian Journal of Genetics, 1996, 32(12): 1417–1430].

*Petrova NA, Michailova PV (1996a) Three-year cytological research of *Chironomusbalatonicus* from Chernobyl zone (1987–1989). In: Shobanov NA, Zinchenko TD (Eds) Ecology, Evolution and Systematics of Chironomidae. Togliatti, Borok, pp. 18–23. [In Russian].

*Petrova N, Michailova P (1996b) Cytogenetic monitoring of *Chironomusbalatonicus* (Diptera, Chironomidae) from the Chernobyl region. International Journal of Dipterological Research 7(2): 79–86.

### ﻿1997

Il’inskaya NB, Petrova NA (1997) Karyotype and inversion polymorphism of natural populations of *Camptochironomustentans* from the North-Western region of Russia (Diptera, Chironomidae). Tsitologiya 39(9): 848–856. [In Russian with English summary].

### ﻿1998

*Michailova P, Petrova N, Sella G, Ramella L, Bovero S (1998) Structural-functional rearrangements in chromosome G in *Chironomusriparius* (Diptera, Chironomidae) collected from a heavy metal-polluted area near Turin, Italy. Environmental Pollution 103: 127–134. https://doi.org/10.1016/S0269-7491(98)00085-2

### ﻿1999

Chubareva LA, Petrova NA (1999) Karyotypic features and rank of supraspecific taxa in Palaearctic blackflies of the subfamily Prosimuliinae Enderlein (Diptera, Simuliidae). Entomologicheskoe Obozrenie 78(1): 189–195. [In Russian with English translation in Entomological Review, 1999, 79(1): 50–57].

Il’inskaya NB, Petrova NA, Matena I (1999) The relationship between the level of inversion polymorphism and the type of water body, the season, and the year of observation in *Chironomusplumosus* L. (Diptera, Chironomidae). Genetika 35(8): 1061–1070. [In Russian with English translation in Russian Journal of Genetics, 1999, 35(8): 908–917].

Petrova NA, Chubareva LA (1999) Karyotype of prodiamesins [sic!] (Diptera, Chironomidae, Prodiamesinae) from the Bratsk Reservoir. Tsitologiya 41(1): 101–103. [In Russian with English summary].

Petrova NA, Chubareva LA, Kachvoryan E (1999) Chromosomal polymorphism in *Chironomusriparius* Meigen (Diptera, Chironomidae) from a southern peripheral population (the Armenian Upland). Tsitologiya 41(12): 1032–1037. [In Russian with English summary].

### ﻿2000

Michailova P, Petrova N, Bovero S, Cavicchioli O, Ramella L, Sella G (2000) Effect of environmental pollution on the chromosomal variability of *Chironomusriparius* Meigen, 1804 (Diptera, Chironomidae) larvae from two Piedmont stations. Genetica 108: 171–180. https://doi.org/10.1023/A:1004172019131

Petrova N, Michailova P, Bovero S (2000) Cytogenetic characteristics of *Chironomusnuditarsis* Str. (Chironomidae, Diptera) and its relationship with species from the plumosus group. In: Hoffrichter O (Ed.) Late 20^th^ Century Research on Chironomidae. An Anthology from the 13^th^ International Symposium on Chironomidae, Freiburg, 5–9 September 1997, Aachen: Shaker Verlag, pp. 201–208.

*Petrova NA, Michailova PV, Sella G, Ramella L, Bovero S, Zelano V, Regoli F (2000) Structural-functional alterations of polytene chromosomes of *Chironomusriparius* from some heavy metal-polluted water bodies of Italy. Sibirskii Ekologicheskii Zhurnal (Siberian Ecological Journal) 4: 511–521. [In Russian with English summary].

### ﻿2001

*Michailova P, Ilkova J, Petrova N, White K (2001a) Rearrangements in the salivary gland chromosomes of *Chironomusriparius* Mg. (Diptera, Chironomidae) following exposure to lead. Caryologia 54(4): 349–363. https://doi.org/10.1080/00087114.2001.10589246.

*Michailova P, Petrova N, Sella G, Bovero S, Ramella L, Regoli F, Zelano V (2001b) Genotoxic effects of chromium on polytene chromosomes of *Chironomusriparius* Meigen, 1804 (Diptera, Chironomidae). Caryologia 54(1): 59–71. https://doi.org/10.1080/00087114.2001.10589213

Petrova NA, Klishko OK (2001) Atypical puffing of *Chironomusplumosus* (Diptera, Chironomidae) in natural population from Chita Region. Tsitologiya 43(2): 172–177. [In Russian with English summary].

Rakisheva AZh, Petrova NA, Michailova P (2001) Larval morphology and karyotypic characteristics of *Chironomusjonmartini* Lindeberg (Diptera, Chironomidae) from peripheral southern population (Mountain Kazakhstan). Entomologicheskoe Obozrenie 80(2): 512–517. [In Russian with English translation in Entomological Review, 2001, 81(9): 1079–1085].

### ﻿2002

*Petrova N, Michailova P (2002) Cytogenetic characteristics of *Chironomusbernensis* Klötzli (Diptera, Chironomidae) from a heavy metal polluted station in Northern Italy. Annales Zoologici 52(2): 227–233.

### ﻿2003

Chubareva LA, Petrova NA (2003) Karyotypes of blackflies (Diptera, Simuliidae) of the world. Entomologicheskoe Obozrenie 82(1): 157–222. [In Russian with English translation in Entomological Review, 2003, 83(2): 149–204].

Chubareva LA, Petrova NA, Kachvoryan EA (2003) Morphological diversity of centromere regions in polytene chromosomes of blackflies (Diptera, Simuliidae). Tsitologiya 45(4): 368–376. [In Russian with English summary].

Kachvoryan EA, Petrova NA, Chubareva LA (2003) The results of karyological study of black flies (Diptera, Simuliidae) in Armenia. Parazitologiya 37(2): 89–102. [In Russian with English summary].

Karageuzyan KG, Chubareva LA, Kachvoryan EA, Adler PH, Petrova NA, Kyureghyan TN, Harutyunova LD, Hovhannisyan VS, Simonyan MA (2003) Ecological conditions of Hrazdan river (Armenia). Report I. Vestnik IAELPS, 8(4): 30–34. [In Russian with English summary].

Petrova NA, Chubareva LA, Adler PN [sic!], Kachvoryan EA (2003) Cytogenetic features of blood-sucking blackfly *Wilhelmiaparaequina* Puri (Diptera: Simuliidae) from Armenia. Genetika 39(1): 41–50. [In Russian with English translation in Russian Journal of Genetics, 2003, 39(1): 32–40. https://doi.org/10.1023/A:1022014725940]

Petrova NA, Klishko OK (2003) Cytogenetic peculiarities of three *Chironomus* species of plumosus group (Diptera, Chironomidae) from Chita Province of Russia. In: Shobanov NA (Ed.) New Data in Chironomidology. Borok: Institute for Biology of Inner Waters of the Russian Academy of Sciences, pp. 64–73. [In Russian with English summary].

Petrova NA, Rakisheva AZh (2003) Karyotype and morphology of the larvae of *Chironomusanthracinus* Zett. (Diptera, Chironomidae) from Eastern Kazakhstan. Tsitologiya 45(4): 428–433. [In Russian with English summary].

Petrova NA, Zelentsov NI, Klishko OK, Chubareva LA (2003) First description of polytene chromosomes, larval morphology and biology of two species of the genus *Propsilocerus* (Diptera, Chironomidae, Orthocladiinae). Trudy Russkogo Entomologicheskogo Obshchestva (Proceedings of the Russian Entomological Society) 74: 33–50. [In Russian]

Vinogradova EB, Petrova NA (2003) Synanthropization in chironomids: *Chironomusriparius* (Diptera, Chironomidae) as an example. Trudy Zoologicheskogo Instituta RAS (Proceedings of the Zoological Institute of Russian Academy of Sciences) 299: 187–196.

Vinokurova NV, Veremeichik YaV, Petrova NA (2003) Aberrations of polytene chromosomes [in] larvae [of] *Chironomusplumosus* from the Lake Shkol’noe in Kaliningrad. In: Shobanov NA (Ed.) New Data in Chironomidology. Borok: Institute for Biology of Inner Waters of the Russian Academy of Sciences, pp. 54–59. [In Russian with English summary].

### ﻿2004

Chubareva LA, Petrova NA (2004) Characteristic features of karyotypes in common species of blackflies (Diptera, Simuliidae) from the northwestern region of Russia. Zoologicheskii Zhurnal 83(11): 1341–1352. [In Russian with English translation as: Karyotypes in common blackfly species (Diptera, Simuliidae) of the northwestern region of Russia, in Entomological Review, 2004, 84(8): 866–877.]

Michailova P, Petrova N (2004) Natural hybridization in insects, Diptera (model group – family Chironomidae). In: Bakerdzhieva N, Michailova P (Eds) Evolution and Ecology – 2004. Seminar Dedicated to the 60^th^ Anniversary of the Union of Scientists in Bulgaria. Proceedings. Sofia, pp. 9–19.

Petrova NA, Chubareva LA, Kachvoryan EA (2004) The karyotypical peculiarities and inversion polymorphism of *Prodiamesaolivacea* Mg. (Diptera, Chironomidae, Prodiamesinae) from Armenia. Vestnik IAELPS, 9(3): 26–29. [In Russian with English summary].

*Petrova NA, Michailova P, Ilkova J (2004) Comparative cytogenetic variation of the salivary gland polytene chromosomes in *Chironomusriparius* Mg., 1804 (Diptera, Chironomidae) from two polluted biotopes of Bulgaria and Russia. Genetika 40(1): 49–58. [In Russian with English translation in Russian Journal of Genetics, 2004, 40: 40–48. https://doi.org/10.1023/B:RUGE.0000013447.90957.1a]

Petrova NA, Michailova PV, Chubareva LA, Shobanov NA, Zelentsov NI (2004) The system of A.A. Chernovskii [1949] as a basis for the cytotaxonomy of the family Chironomidae. Euroasian Entomological Journal 3(4): 253–258. [In Russian with English summary]

Sella G, Bovero S, Ginepro M, Michailova P, Petrova N, Robotti CA, Zelano V (2004) Inherited and somatic cytogenetic variability in Palearctic populations of *Chironomusriparius* Meigen, 1804 (Diptera, Chironomidae). Genome 47: 332–344. https://doi.org/10.1139/g03-128.

Vinogradova EB, Petrova NA (2004) First record of a synanthropic population of *Chironomusriparius* Meigen, 1804 (Diptera, Chironomidae) in dwelling house basements in St. Petersburg and some of its biological and karyological characteristics. Entomologicheskoe Obozrenie 83(2): 334–348. [In Russian with English translation in Entomological Review, 2004, 84(7): 752–763.]

### ﻿2005

Michailova P, Petrova N (2005) Comparative effect of heavy metals on the polytene chromosomes of Chironomidae, Diptera. In: Gruev B, Nikolova M, Donev A (Eds) Proceedings of the Balkan Scientific Conference of Biology in Plovdiv (Bulgaria) from 19^th^ till 21^st^ of May 2005, pp. 539–552.

Petrova NA, Klishko OK (2005) Cytodiagnosis, inversion polymorphism, and B-chromosomes of three *Chironomus* sibling species of the group plumosus (Diptera, Chironomidae) from Eastern Siberia. Zoologicheskii Zhurnal 84(7): 838–849. [In Russian with English translation in Entomological Review, 2005, 85(7): 729–740.]

### ﻿2006

Chubareva LA, Petrova NA (2006) B chromosome polymorphism of blackflies (Diptera, Simuliidae) from the north-western region of Russia. Tsitologiya 48(3): 258–263. [In Russian with English summary].

Michailova P, Petrova N, Ilkova J, Bovero S, Brunetti S, White K, Sella G (2006) Genotoxic effect of copper on salivary gland polytene chromosomes of *Chironomusriparius* Meigen, 1804 (Diptera, Chironomidae). Environmental Pollution 144: 647–654. https://doi.org/10.1016/j.envpol.2005.12.041

Petrova NA, Chubareva LA (2006) Karyotypes of Siberian and Far-Eastern blackflies (Diptera, Simuliidae). Euroasian Entomological Journal 5(2): 111–121. [In Russian with English summary].

### ﻿2007

Chubareva LA, Petrova NA (2007) Karyological characters of blackflies (Diptera: Simuliidae). Comparative Cytogenetics 1(1): 89–94.

Chubareva LA, Petrova NA, Reva MV (2007) Karyotypic and morphological study of five species of the genus *Wilhelmia* Enderlein (Diptera, Simuliidae). Entomologicheskoe Obozrenie 86(6): 895–904. [In Russian with English translation in Entomological Review, 2007, 87(9): 1290–1299.]

Ilkova J, Hankeln T, Schmidt ER, Michailova P, Petrova N, Sella G, White K (2007) Genome instability of *Chironomusriparius* Mg. and *Chironomuspiger* Strenzke (Diptera, Chironomidae). Caryologia 60(4): 299–308. https://doi.org/10.1080/00087114.2007.10797951

Kachvoryan EA, Oganesyan VS, Petrova NA, Zelentsov NI (2007) The fauna of chironomids and blackflies (Diptera: Chironomidae, Simuliidae) and hydrochemical characteristics of the Hrazdan River (Armenia). Entomologicheskoe Obozrenie 86(1): 104–113. [In Russian with English translation in Entomological Review, 2007, 87(1): 73–81. https://doi.org/10.1134/S0013873807010071]

Michailova P, Ilkova J, Petrova N, Selvaggi A, Zampicinini GP, Sella G (2007) The relationship between chromosome rearrangements and repetitive DNA clusters in *Chironomusriparius* Meigen (Diptera: Chironomidae) from anthropogenically polluted Palaearctic regions. Comparative Cytogenetics 1(1): 45–49.

Michailova P, Sella G, Petrova N (2007) Effect of heavy metals on the genome of model insect group (Diptera, Chironomidae). In: Evolution and Ecology – 2007. Proceedings. Sofia, pp. 36–43.

Petrova NA, Chubareva LA, Reva MV (2007) Cytogenetic analysis of the noxious bloodsucker *Boophthoraerythrocephala* (Diptera: Simuliidae) from different geographic zones. Tsitologiya 49(4): 329–339. [In Russian with English summary].

Petrova NA, Vinokurova NV, Danilova MV (2007) Chromosomal variation of populations of *Chironomusplumosus* Linnaeus (Diptera: Chironomidae) from lakes of Kaliningrad, Russia. Comparative Cytogenetics 1(1): 51–54.

Petrova NA, Vinokurova NV, Danilova MV, Maslova VV (2007) Seasonal variability of the karyotype structure of *Chironomusplumosus* (Diptera, Chironomidae) from a biotope of Kaliningrad. Tsitologiya 49(10): 901–905. [In Russian with English summary].

### ﻿2008

*Chubareva LA, Petrova NA (2008) Cytogenetic maps of polytene chromosomes and some morphological peculiarities of blackflies from Russia and adjacent territories (Diptera, Simuliidae). Atlas. St. Petersburg, Moscow: KMK Scientific Press, 135 pp., 218 plates. [In Russian].

Ilkova J, Michailova P, Petrova N, Sella G, Hankeln T, Schmidt E (2008) Repetitive DNA localization in two homosequential species of the genus *Chironomus* Kieffer (Diptera, Chironomidae) and their genome reaction to anthropogenic factors. Acta Zoologica Bulgarica, Supplement 2: 41–48.

Petrova NA, Zhirov SV (2008) Cytogenetics of *Chironomusriparius* L. from the fishpond in Borok village (Diptera, Chironomidae, Diptera). Tsitologiya 50(6): 535–538. [In Russian with English summary].

Petrova NA, Zhirov SV (2008) Polytene chromosomes of salivary glands of chironomids (Diptera: Chironomidae) from the Wrangel Island (Russia). Comparative Cytogenetics 2(2): 127–130.

### ﻿2009

Chubareva LA, Petrova NA (2009) Karyotypes of rare and little-known species of blackflies (Diptera, Simuliidae). Acta Zoologica Lithuanica 19(3): 175–177. https://doi.org/10.2478/v10043-009-0022-7

Petrova NA, Zhirov SV (2009) Inversion polymorphism in two chironomid species of the genera *Chironomus* and *Camptochironomus* (Diptera, Chironomidae, Chironomini) from different regions of Russia (central part and North-West). Vestnik Sankt-Peteburgskogo Gosudarstvennogo Universiteta, Series 3, Biologiya, 4: 29–39, 149–150, 154–155. [In Russian]

### ﻿2010

Michailova P, Petrova N, Sella G (2010) Genome response of model insect group (ChironomidaeDiptera) to trace metal contaminants in the environment. In: Recent Advances in Mathematics and Computers in Business, Economics, Biology and Chemistry. Proceedings of the 11^th^ WSEAS International Conference on Mathematics and Computers in Business and Economics (MCBE’10). “G. Enescu” University, Iasi, Romania, June 13–15, 2010, pp. 366–372.

Michailova P, Petrova N, Sella G, Bovero S, White K, Ramella L (2010) Cytogenetic biomarkers in *Chironomusriparius* Mg. (Diptera) as indicators of heavy metal pollution. In: Ferrington LC, Jr (Ed.) Proceedings of the XV International Symposium on Chironomidae. Saint Paul, Minnesota: University of Minnesota, pp. 235–242.

Petrova NA, Zhirov SV (2010) Larvae morphology, karyotype structure, and inversion polymorphism in a chironomid from the Republic of South Africa (Diptera, Chironomidae). Vestnik VOGiS 14(1): 70–78. [In Russian with English summary].

Sharton AYu, Petrova NA, Vinokurova NV, Danilova MV, Zolotova SM (2010) Inversion polymorphism of *Glyptotendipesglaucus* Mg. (Diptera: Chironomidae) from the reservoirs of Kaliningrad. Genetika 46(7): 887–895. [In Russian with English translation in Russian Journal of Genetics, 2010, 46(7): 786–793. https://doi.org/10.1134/S1022795410070021]

### ﻿2011

Michailova P, Sella G, Petrova N (2011) Chironomids (Diptera) and their salivary gland chromosomes as indicators of trace-metal genotoxicity. Italian Journal of Zoology 79(2): 218–230. https://doi.org/10.1080/11250003.2011.622084

Petrova NA, Vinokurova NV, Danilova MV, Sharton AYu (2011) Inversion polymorphism in the population of *Camptochironomustentans* from Kaliningrad city. Tsitologiya 53(7): 580–585. [In Russian with English summary].

Petrova NA, Zhirov SV (2011) Cytogenetic comparison of chironomid midge *Glyptotendipesglaucus* (Meigen, 1818) (Diptera, Chironomidae) populations from northwest Russia and Ukraine (Chernobyl zone). Ekologicheskaya Genetika (Ecological Genetics) 9(2): 9–16. [In Russian with English summary] https://doi.org/10.17816/ecogen1029-16

Petrova NA, Zhirov SV, Zelentsov NI, Kachvoryan EA (2011) To the fauna of chironomids (Diptera, Chironomidae) from the Razdan River valley (Armenia). Zoologicheskii Zhurnal 90(4): 445–451. [In Russian with English translation as: On the fauna of Chironomidae (Diptera) of the Hrazdan Basin (Ar­menia), in Entomological Review 91(3): 360–366. https://doi.org/10.1134/S0013873811030110]

### ﻿2012

*Michailova P, Sella G, Petrova N (2012) Polytene chromosomes of Chironomidae (Diptera) as a bioassay of trace-metal-induced genome instability. In: Ekrem T, Stur E, Aagaard K (Eds) Proceedings of the 18^th^ International Symposium on Chironomidae. Fauna Norvegica 31: 227–234. https://doi.org/10.5324/fn.v31i0.1355

Petrova NA, Zhirov SV, Harutyunova KV, Harutyunova MV (2012) Morphological deformations of mouth parts in some species of the subfamilies Orthocladiinae and Diamesinae (Diptera, Chironomidae). Biological Journal of Armenia, 64(4): 48–52. [In Russian with English summary].

Petrova NA, Zhirov SV, Harutyunova MV, Harutyunova KV (2012) Cytotaxonomy and morphology of chironomid larvae (Diptera, Chironomidae) in Armenia. World Academy of Science, Engineering and Technology, 65: 513–517.

### ﻿2013

*Petrova NA (2013) Reorganization of polytene chromosomes of chironomid larvae (Diptera, Chironomidae) as a response to mutagenic pollution of environment (Chernobyl ecocatastrophe). St. Petersburg: Zoological Institute of Russian Academy of Sciences, 98 pp. [In Russian].

*Petrova NA, Michailova PV, Bovero S, Sella G (2013) Karyotypes of four species of chironomids (Diptera, Chironomidae) from Northern Italy. Tsitologiya 55(6): 436–441. [In Russian with English translation in Cell and Tissue Biology, 2013, 7(5): 465–471. https://doi.org/10.1134/S1990519X13050088]

Zhirov SV, Petrova NA (2013) The chironomid midge *Dicrotendipes* sp. afr. (Diptera, Chironomidae) from the Republic of South Africa. Zoologicheskii Zhurnal 92(4): 464–471. [In Russian with English translation in Entomological Review, 2013, 93(6): 695–702. https://doi.org/10.1134/S0013873813060031]

Petrova NA, Zhirov SV (2013) Characteristics of the karyotypes of three subfamilies of chironomids (Diptera, Chironomidae: Tanypodinae, Diamesinae, Prodiamesinae) of the world fauna. Entomologicheskoe Obozrenie 92(3): 505–516. [In Russian with English translation in Entomological Review, 2013, (2014) 94(2): 157–165. https://doi.org/10.1134/S001387381402002X]

### ﻿2014

Karmokov MKh, Belyanina SI, Zhirov SV, Petrova NA (2014) Karyotype and morphology of a midge *Stictochironomuscrassiforceps* (Kieffer) (Diptera, Chironomidae) in several parts of the Palaearctic Region. Entomologicheskoe Obozrenie 93(3–4): 555–563. [In Russian with English translation in Entomological Review 94(9): 1229–1238. https://doi.org/10.1134/S0013873814090048]

*Petrova NA, Zhirov SV, Erbaeva EA (2014a) Description of three species of chironomids (Diptera, Chironomidae) from Lake Khubsugul, Mongolia (morphological and karyological aspects). Euroasian Entomological Journal 13(5): 445–450. [In Russian with English summary].

*Petrova NA, Zhirov SV, Harutyunova K, Harutyunova M (2014b) On the possibility of spontaneous interspecific hybridization in the nature of representatives of sibling-species *Chironomusriparius* Kieffer and *Chironomuspiger* Strenzke (Diptera, Chironomidae) from Armenia. Tsitologiya 56(2): 170–174. [In Russian with English summary].

### ﻿2015

Lobkova LE, Orel OV, Zhirov SV, Petrova NA (2015) Chironomus (Chironomus) acidophilus Keyl, 1960 (Diptera, Chironomidae, Chironominae): biology, morphology, karyotype and habitat conditions in the caldera of the Uzon Volcano (Kamchatka, Kronotsky Nature Reserve). In: Lobkov EG (Ed.) Trudy Kronotskogo Gosudarstvennogo Prirodnogo Biosfernogo Zapovednika, 4: 92–119, 176. Petropavlovsk-Kamchatskii: Kamchatpress. [In Russian with English summary].

*Michailova P, Petrova NA (2015) Bioindicator potential of cytogenetic variability in polytene chromosomes of chironomids (Diptera, Chironomidae) to assess environmental pollution. Tsitologiya i Genetika 49(4): 61–70. [In Russian with English translation in Cytology and Genetics, 2015, 49(4): 262–269. https://doi.org/10.3103/S0095452715040064]

Orel OV, Lobkova LE, Zhirov SV, Petrova NA (2015) A new record of Chironomus (Chironomus) acidophilus Keyl (Diptera, Chironomidae) from the Uzon volcanic caldera (Kronotsky Reserve, Kamchatka Peninsule, Russia), its karyotype, ecology and biology. Zootaxa 3981(2): 177–192. https://doi.org/10.11646/zootaxa.3981.2.2

*Petrova NA, Cornette R, Shimura S, Gusev O, Pemba D, Kikawada T, Zhirov S, Okuda T (2015) Karyotypical characteristics of two allopatric African populations of anhydrobiotic *Polypedilum* Kieffer, 1912 (Diptera, Chironomidae) originating from Nigeria and Malawi. Comparative Cytogenetics 9(2): 173–188. https://doi.org/10.3897/CompCytogen.v9i2.9104

Petrova NA, Zhirov SV (2015) The cytogenetic characteristic of some Palearctic populations of Holarctic midge *Glyptotendipesbarbipes* Staeger (Diptera, Chironomidae). Tsitologiya 57(11): 831–837. [In Russian with English summary].

Zhirov SV, Petrova NA (2015) Karyotypes and larval morphology of three species of midges (Diptera, Chironomidae) from lakes in the southern part of Kunashir Island. Entomologicheskoe Obozrenie 95(3): 599–607. [In Russian with English translation in Entomological Review, 2015, 95(7): 881–890. https://doi.org/10.1134/S0013873815070064]

### ﻿2016

Petrova NA, Zhirov SV (2016) The role of salivary gland polytene chromosomes of chironomid (Diptera, Chironomidae) larvae in species identification and bioindication of environment pollution. In: X All-Russian Dipterolical [sic!] Symposium (with International Participation), Krasnodar, 23–28 August 2016. Materials. Krasnodar: Kuban State University, pp. 267–274. [In Russian with English summary].

### ﻿2017

*Cornette R, Yamamoto N, Yamamoto M, Kobayashi T, Petrova NA, Gusev O, Shimura S, Kikawada T, Pemba D, Okuda T (2017) A new anhydrobiotic midge from Malawi, *Polypedilumpembai* sp. n. (Diptera: Chironomidae), closely related to the desiccation tolerant midge, *Polypedilumvanderplanki* Hinton. Systematic Entomology 42: 814–825. https://doi.org/10.1111/syen.12248

Petrova NA, Zhirov SV (2017) Karyotype characteristics of *Chironomusfraternus* Wülker and *Ch.beljaninae* Wülker (Diptera, Chironomidae) from Northern Russia. Entomologicheskoe Obozrenie 96(3): 429–435. [In Russian with English translation in Entomological Review, 2017, 97(6): 730–734. https://doi.org/10.1134/S0013873817060033]

### ﻿2018

Przhiboro A, Saidov A, Petrova N (2018) Firuz Akhrorov (1937–2012) and his contributions to the study of Chironomidae in Tajikistan. In: Lencioni V, Cranston PS, Makarchenko EA (Eds) Recent advances in the study of Chironomidae: An overview. Journal of Limnology 77(s1): 10–14. https://doi.org/10.4081/jlimnol.2018.1800

### ﻿2019

*Kuznetsova V, Golub N, Petrova N, Lukhtanov V, Anokhin B, Khabasova N, Shapoval N, Kupriyanova L, Gavrilov-Zimin I (2019) In Memoriam: Dr. Sergey V. Zhirov (1966–2017). Comparative Cytogenetics 13(3): 321–324. https://doi.org/10.3897/CompCytogen.v13i3.47366

Petrova NA, Zhirov SV, Krasheninnikov AB (2019) Larval morphology and karyotype structure of *Chironomus* sp. (Diptera, Chironomidae) from the Novaya Zemlya Archipelago (Russia). Entomologicheskoe Obozrenie 98(4): 761–771. https://doi.org/10.1134/S0367144519040099 [In Russian with English translation in Entomological Review, 2019, 99(8): 1183–1191. https://doi.org/10.1134/S0013873819080128]

### ﻿2020

Markiyanova MF, Petrova NA (2020) Chromosomal variability in *Chironomusplumosus* (Linnaeus, 1758) (Diptera, Chironomidae) from a coastal lagoon of the Baltic Sea (Curonian Lagoon). Zoologicheskii Zhurnal 99(9): 1002–1013. https://doi.org/10.31857/S0044513420090135 [In Russian with English translation in Entomological Review, 2020, 100(7): 969–981. https://doi.org/10.1134/S0013873820070027]

Petrova NA (2020) Chromosome evolution in Chironomidae (Diptera, Chironomidae). In: Ovchinnikova OG, Shamshev IV (Eds) XI All-Russian Dipterological Symposium (with international participation), Voronezh, 24–29 August 2020. Materials of St. Petersburg: Russian Entomological Society: LEMA Publ., pp. 188–192. [In Russian with English summary]. https://doi.org/10.47640/978-5-00105-586-0_2020_188

### ﻿2021

*Petrova NA (2021) Ninel Alekseevna Petrova. In: Brodskaya NK, Dunaeva YuA, Przhiboro AA, Tikhonova EP (Eds.) War’s Tragic Memory: The Great Patriotic War in the Memoirs of the Staff of the Zoological Institute of the Russian Academy of Sciences. St. Petersburg: Russkaya kollektsiya, pp. 322–325. [In Russian].

Petrova NA, Michailova PV (2021) The use of salivary gland chromosomes of chironomids (Diptera, Chironomidae) for assessing the pollution of aquatic ecosystems. Euroasian Entomological Journal 20(1): 38–48. [In Russian with English summary]. https://doi.org/10.15298/euroasentj.20.1.06

### ﻿2022

*Petrova NA, Zhirov SV (2022) Structure of polytene chromosomes and larval morphology of chironomids (Diptera, Chironomidae). Atlas. St. Petersburg, Moscow: KMK Scientific Press, 114 pp., 155 plates. [In Russian].

### ﻿2024

Petrova NA (2024) B-chromosomes of Chironomidae and Simuliidae (Diptera): a brief review. Euroasian Entomological Journal 23(4): 209–214. [In Russian with English summary]. https://doi.org/10.15298/euroasentj.23.04.04

## Supplementary materials

Supplementary material 1 A list of publications by Ninel Petrova (compiled by A. Przhiboro) Data type docx https://binary.pensoft.net/file/1202123This dataset is made available under the Open Database License (http://opendatacommons.org/licenses/odbl/1.0/). The Open Database License (ODbL) is a license agreement intended to allow users to freely share, modify, and use this Dataset while maintaining this same freedom for others, provided that the original source and author(s) are credited.Valentina Kuznetsova, Paraskeva Michailova, Andrey Przhiboro, Natalia Khabazova

