# Supplementary material for: ﻿In Memoriam: Cytogeneticist Dr. Sc. Ninel A. Petrova (1940–2024) — life and scientific heritage
Source: Comp Cytogenet. 2024 Dec 18;18:277–305. doi: 10.3897/compcytogen.18.138747 (PMC11669904; doi:10.3897/compcytogen.18.138747)
Supplement: Supplementary material 1 — A list of publications by Ninel Petrova (compiled by A. Przhiboro) [file comparative_cytogenetics-18-277_article-138747__-s001.docx]

**A list of publications by Ninel Petrova (compiled by A. Przhiboro)**

Comments. The titles many articles published in Russian differ significantly in content from the English translations of these titles given in the same article. In such cases, we give only the English titles of the corresponding articles (comments and additions are given in brackets, if necessary). For articles published as English translations after the original Russian publications, the title of the article is given only if it differs significantly from the original. The spelling of the surnames of N.B. Il’inskaya, I.A. Rubtsov and N.I. Zelentsov is everywhere given as here.

1967

Petrova NA, Fedorov VS (1967) Genetics of rye (*Secale cereale* L.). VII. Cytoembryological characteristics of diploid and tetraploid rye forms in case of their reciprocal hybridization. Vestnik Leningradskogo Universiteta (Bulletin of Leningrad State University) 9(2): 145–152. [In Russian with English summary]

1968

Chubareva LA, Petrova NA (1968) Homologous lines of chromosome polymorphism in the natural populations of blackflies (Diptera, Simuliidae). Tsitologiya 10(10): 1248–1256. [In Russian with English summary]

1969

Chubareva LA, Petrova NA (1969) Karyological peculiarities of *Helodon ferrugineus* Wahlb. in relation to some questions of systematics. Tsitologiya 11(2): 234–241. [In Russian with English summary]

1970

Chubareva LA, Petrova NA (1970) Structural features of polytene chromosomes and their use in taxonomic diagnostics of the family Simuliidae. In: 2nd Symposium on Structure and Functioning of Chromosomes. Abstracts. Novosibirsk, 7–13 January 1970, pp. 76–77. [In Russian].

Petrova NA, Rubtsov IA, Chubareva LA (1970) Morphological and karyological characters in the systematics of *Schönbaueria gigantea* (Diptera, Simuliidae). In: Annual scientific session summarizing research in 1969, 23–25 March 1970. Abstracts. Leningrad: Zoological Institute of the Academy of Sciences of the USSR, pp. 23–24. [In Russian].

1971

Chubareva LA, Petrova NA, Petrukhina TE (1971) The structural peculiarities of the polytene chromosomes and the taxonomy of the black flies (Diptera, Simuliidae). Tsitologiya 13(6): 784–789. [In Russian with English summary]

Petrova NA, Rubtsov IA, Chubareva LA (1971) On the position of *Simulium* (*Schönbaueria*) *gigantea* Rubz. in the system of classification of simuliids (Morphological and karyological characters in the systematics). Parazitologiya 5(1): 40–50. [In Russian; English translation: Parasitology (1972) 1(1): 45–57.]

1972

Petrova NA (1972) Karyological features of black flies of the genus *Cnephia* End. In: Annual scientific session summarizing research in 1971, 20–22 March 1972. Abstracts. Leningrad: Zoological Institute of the Academy of Sciences of the USSR, p. 23. [In Russian]

Petrova NA (1972) Karyological features of the Karelian black flies [sic!] of the genus *Cnephia* End. Tsitologiya 14(6): 769–773, 2 plates. [In Russian with English summary]

1973

Petrova NA (1973) A comparative karyological analysis of three genera of the family Simuliidae (Diptera). Tsitologiya 15(8): 1055–1059. [In Russian with English summary]

Petrova NA (1973) A comparative karyological study of 3 species of black-flies of the genus *Metacnephia* Crosskey (Diptera, Simuliidae) from Armenia. Tsitologiya 15(4): 439–445, 3 plates. [In Russian with English summary]

1974

Petrova NA (1974) Inversional polymorphism in natural populations of two species of black flies (Diptera, Simuliidae). Genetika 10(1): 80–89. [In Russian with English summary]

1975

Chubareva LA, Petrova NA (1975) Karyotype of the plesiomorphic New Zealand species *Austrosimulium tillyardi* and its genetical relations with some other genera of the family Simuliidae (Diptera). Zoologicheskii Zhurnal 54(4): 552–558. [In Russian with English summary]

Petrova NA (1975) Comparative karyological study of blood-sucking black flies of the genera *Cnephia* End., *Metacnephia* Crossk. and *Sulcicnephia* Rubz. (Diptera, Simuliidae). Abstract of Candidate of Biological Sciences Dissertation, Leningrad: Zoological Institute, Academy of Sciences of the USSR, 20 pp. [In Russian]

Petrova NA (1975) Comparative karyological study of blood-sucking black flies of the genera *Cnephia* End., *Metacnephia* Crossk. and *Sulcicnephia* Rubz. (Diptera, Simuliidae). Candidate of Biological Sciences Dissertation, Leningrad: Zoological Institute, Academy of Sciences of the USSR, 242 pp. [In Russian]

1976

Chubareva LA, Rubtsov IA, Petrova NA (1976) Morphological and karyological similarities and differences in Palearctic and Neotropical species of the genus *Hemicnetha* End. (Diptera, Simuliidae). Entomologicheskoe Obozrenie 55(2): 452–457. [In Russian; English translation: Entomological Review 55(2): 137–142.]

1977

Petrova NA (1977) A new species of black flies; *Metacnephia pamiriensis* sp. n. (Simuliidae) from Pamir. Parazitologiya 11(3): 210–212, 1 plate. [In Russian with English summary]

Petrova NA, Chubareva LA, Kuzmenko KN (1977) The karyotypes of five species of Chironomidae (Diptera). Tsitologiya 19(8): 900–905. [In Russian with English summary]

Petrova NA, Maksimova FL (1977) Chromosomal transformations in natural populations of *Chironomus plumosus* L. (Diptera). In: 3rd Congress of NI Vavilov All-Union Society of Geneticists and Breeders, Leningrad, 16–20 May 1977. Abstracts. Leningrad: Nauka, p. 346. [In Russian]

Rubtsov IA, Petrova NA (1977) Blackflies of the tribe Cnephiini (Diptera, Simuliidae) and diagnoses of the genera *Cnephia* Enderlein and *Astega* Enderlein. Entomologicheskoe Obozrenie 56(3): 691–697. [In Russian; English translation: Entomological Review 56(3): 145–149.]

1978

Maksimova FL, Petrova NA (1978) Geographical variability of karyotype in *Chironomus plumosus* (Diptera, Chironomidae). Zoologicheskii Zhurnal 57(12): 1816–1826. [In Russian with English summary]

Petrova NA (1978) Karyologic study of Orthocladiinae (Diptera, Chironomidae). In: Belyaev DK (Ed.) XIV International Congress of Genetics. Moscow, 21–30 August 1978. Sectional sessions. Abstracts. Part 1. Sections 1–12, Moscow: Nauka, p. 271. [In Russian]

Petrova NA, Chubareva LA (1978) The peculiarities of the karyotype of *Prodiamesa olivacea* Meig. (Diptera, Chironomidae, Orthocladiinae). Tsitologiya 20(10): 1208–1211. [In Russian with English summary]

Petrova NA, Maksimova FL (1978) The role of chromosome rearrangements in the speciation of chironomids (Diptera, Chironomidae). Genetika 14(7): 1201–1207. [In Russian; English translation: Soviet Genetics 14: 849–853.]

1979

Chubareva LA, Petrova NA (1979) Main characters of karyotypes of blackflies (Diptera, Simuliidae) of the world. In: Chubareva LA (Ed.) Karyosystematics of the Invertebrate Animals. Leningrad: Zoological Institute, Academy of Sciences of the USSR, pp. 58–95. [In Russian with English summary]

Petrova NA (1979) Chromosomal rearrangements distinguish species of the genus *Metacnephia* (Diptera, Simuliidae). In: Nartshuk EP (Ed.) Ecological and Morphological Principles of Diptera Systematics (Insecta). Materials of Symposium (13–15 September 1978, Voronezh). Leningrad: Zoological Institute AN SSSR, pp. 79–81. [In Russian: English translation: Skarlato OA (Ed.) Systematics of Diptera (Insecta): Ecological and Morphological Principles. New Delhi, India: Amerind Publishing Co. Pvt. Ltd., pp. 125–128.]

Petrova NA, Chubareva LA, Zolotaryova LV, Kaliberdo TA (1979) The karyotypic study of natural populations of two chironomid species from the Bratsk Reservoir (Diptera). In: Kozhova OM (Ed.) Problems of ecology of the Baikal region (Abstracts of reports for the republican meeting. Irkutsk, 10–13 September 1979). II. Ecological and genetic questions of functioning of natural systems. III. Mathematical modelling of ecosystems and populations. Irkutsk, pp. 50–51. [In Russian]

1980

Chubareva LA, Petrova NA (1980) A method for making of cytological preparations for caryological study of Diptera. In: Chubareva LA (Ed.) New Data on Karyosystematics of Dipterous Insects. Trudy Zoologicheskogo Instituta AN SSSR, 95: 73–80. [In Russian with English summary; signed for press on 29 December 1980 and possibly therefore actually published in 1981]

Maksimova FL, Petrova NA (1980) On the problem of sympatric species evolution in chironomids (Diptera, Chironomidae). In: Lukina EV (Ed.) Problems of Population Cytogenetics of Plants and Animals, pp. 70–76. [In Russian]

Pankratova VYa, Chubareva LA, Petrova NA (1980) On the systematics of some *Chironomus* species (Chironomidae) from the Lake Sevan. In: Chubareva LA (Ed.) New Data on Karyosystematics of Dipterous Insects. Trudy Zoologicheskogo Instituta AN SSSR, 95: 50–54. [In Russian with English summary]

Petrova NA (1980) Cytological sex determination of blackflies (Simuliidae). In: Chubareva LA (Ed.) New Data on Karyosystematics of Dipterous Insects. Trudy Zoologicheskogo Instituta AN SSSR, 95: 55–58. [In Russian with English summary]

Petrova NA (1980) Karyological research of Orthocladiinae (Diptera, Chironomidae). In: Vorontsov NN, van Brink JM (Eds) Animal Genetics and Evolution. Selected Papers of the XIV International Congress of Genetics, 21–30 August 1978, Moscow. Genetica 52–53: 275–279. Hague: Dr W Junk B.V. Publ. https://doi.org/10.1007/BF00121837

1981

Chubareva LA, Petrova NA (1981) A new genus of black flies (Diptera, Simuliidae) from Tadzhikistan. Entomologicheskoe Obozrenie 60(4): 898–900. [In Russian; English translation: Entomological Review (1982) 60(4): 140–144.]

Petrova NA (1981) Morphological peculiarities of polytene chromosomes in two black fly species (Diptera, Simuliidae). Tsitologiya 23(11): 1317–1320. [In Russian with English summary]

Petrova NA (1981) Peculiarities of karyotype of *Syndiamesa nivosa* (Diptera, Chironomidae). In: Bochkov NP (Ed.) The Fourth Congress of NI Vavilov All-Union Society of Geneticists and Breeders. Kishinev, 1–5 February 1982. Abstracts. Part 1, Kishinev: Shtiyintsa, pp. 190–191. [In Russian]

Petrova NA, Chubareva LA, Zolotaryova LV, Kaliberdo TA (1981) The karyotypes of chironomids from the Bratsk Reservoir (Diptera). Tsitologiya 23(10): 1180–1187. [In Russian with English summary]

1982

Chubareva LA, Petrova NA (1982) Cytogenetic method of chromosome analysis in chironomids. In: Akhrorov F (Ed.) Methodical Guide to Chironomid Research. Dushanbe: Donish, pp. 64–73. [In Russian]

Petrova NA, Zolotareva LV (1982) Morphology and karyotype of a larva of *Micropsectra* sp. (Diptera, Chironomidae) from the East Pamir. Zoologicheskii Zhurnal 61(10): 1605–1607. [In Russian with English summary]

1983

Belyanina SI, Maksimova FL, Bukhteeva NM, Il’inskaya NB, Petrova NA, Chubareva LA (1983) Chapter 2. Systematics and morphology. Division 2. Karyotype. In: Sokolova NJu (Ed.) *Chironomus plumosus* L. (Diptera, Chironomidae). Systematics, Morphology, Ecology, Production. Moscow: Nauka Publishers, pp. 61–96. [In Russian]

Chubareva LA, Petrova NA (1983) A new species of blackflies of genus *Prosimulium* (Roub.) [sic!] (Simuliidae, Diptera) from Pamir. In: Nartshuk EP (Ed.) Diptera (Insecta), Their Systematics, Geographic Distribution and Ecology (15–17 September 1982, Belaya Tserkov’). Leningrad: Zoological Institute AN SSSR, pp. 141–144. [In Russian]

Petrova NA (1983) A new genus and species of buffalo gnats (Diptera, Simuliidae) from West Pamir. Zoologicheskii Zhurnal 62(12): 1911–1915. [In Russian with English summary]

Petrova N (1983) The karyotype and unstable associations of polytene chromosomes in *Syndiamesa nivosa* (Diptera, Chironomidae). Zoologicheskii Zhurnal 62(1): 69–74. [In Russian with English summary]

Petrova NA (1983) Population variability of blood-sucking black fly *Sulcicnephia ovtchinnikovi* (Simuliidae). Parazitologiya 17(6): 470–473. [In Russian with English summary]

1984

Chubareva LA, Petrova NA (1984) B chromosomes of black flies (Simuliidae, Diptera). Genetika 20(4): 570–578. [In Russian; English translation: Soviet Genetics 20(4): 446–453.]

Michailova P, Petrova N (1984) Initial stage of sympatric divergency in species of the genus *Glyptotendipes* Kieff. (Diptera, Chironomidae). Caryologia 37(4): 293–307. https://doi.org/10.1080/00087114.1984.10797708

Il’inskaya NB, Petrova NA (1984) Results and perspectives of karyological studies of *Chironomus plumosus* L. In: Ol’shvang VN (Ed.) Species and Its Productivity in the Range. Part IV. Invertebrates. Proceedings of the 4th All-Union Meeting, 3–7 April 1984, Sverdlovsk, pp. 55–56. [In Russian]

Petrova NA (1984) Current state of knowledge of karyotypes in chironomids. In: Vasiliev VP (Ed.) IX Congress of the All-Union Entomological Society. Abstracts. Part 2, Kiev: Naukova Dumka, p. 95. [In Russian]

Petrova NA, Chubareva LA (1984) A list of species of the black flies (Diptera, Simuliidae) of Tajikistan. In: Nartshuk EP, Zlobin VV (Eds) Diptera (Insecta) of the Fauna of the USSR and Their Significance in Ecosystems (15–17 September 1982, Belaya Tserkov’). Leningrad: Zoological Institute AN SSSR, pp. 99–106. [In Russian; English translation: Entomological Review (1992) 71(3): 39–46.]

1985

Il’inskaya NB, Petrova NA (1985) B-chromosomes of *Chironomus plumosus* (Diptera, Chironomidae). Genetika 21(10): 1671–1679. [In Russian with English summary]

Petrova NA, Feher LV (1985) Chromosomal polymorphism in *Glyptotendipes paripes* (Edw.) (Diptera, Chironomidae). Tsitologiya 27(6): 710–713. [In Russian with English summary]

1986

Petrova NA (1986) The value of karyological characters for the taxonomy, systematics and evolution of chironomids. In: Kolesnikov NN, Istomina AG (Eds) Evolution, Speciation and Systematics of Chironomids. Novosibirsk: Institute of Cytology and Genetics of the Academy of Sciences of the USSR, pp. 29–35. [In Russian]

Petrova N, Kiknadze I, Michailova P (1986) Integration of species in the plumosus-group of Chironomidae. In: Aukshtikal’nene AM, Permyakova LV (Eds) A System of Species Integration. Vilnius, pp. 138–161. [In Russian with English summary]

Petrova NA, Michailova P (1986) The population karyological studies of some Chironomidae species (Diptera, Chironomidae). Tsitologiya 28(7): 727–734. [In Russian with English summary]

1987

Michailova P, Petrova NA (1987) Peculiarities of the karyotype of *Micropsectra* gr. *notescens* (Diptera, Chironomidae) from different populations. Tsitologiya 29(9): 1056–1060. [In Russian with English summary]

Petrova NA (1987) Chromosome numbers in the Chironomidae. In: Nartshuk EP (Ed.) Diptera and Their Importance for Animal Husbandry and Agriculture (17–19 September 1986, Alma-Ata). Leningrad: Zoological Institute AN SSSR, pp. 136–143. [In Russian]

Petrova NA (1987) Karyological analysis of primitive chironomids (Diptera, Tanypodinae). In: V Congress of NI Vavilov All-Union Society of Geneticists and Breeders, Abstracts. Volume 3, Moscow, p. 123. [In Russian]

1988

Il’inskaya NB, Petrova NA, Dyomin SYu (1988) Seasonal variations of chromosomal polymorphism in *Chironomus plumosus* L. (Diptera, Chironomidae). Genetika 24(8): 1393–1401. [In Russian; English translation: Soviet Genetics 24: 967–974.]

Makarchenko EA, Petrova NA (1988) Chironomids of the subfamily Tanypodinae of the Far East of the USSR. I. Morpho-karyological description of *Macropelopia paranebulosa* Fittkau. In: Levanidova IM, Makarchenko EA (Eds) Fauna, Systematics and Biology of Freshwater Invertebrates. Vladivostok, pp. 28–35. [In Russian]

Petrova NA (1988) Ecological and population study of three species of chironomids of the genus *Glyptotendipes* from the Bratsk Reservoir. In: Problems of Ecology of the Baikal Region. Abstracts the 3rd All-Union Scientific Conference (Irkutsk, 5–10 September 1988), Part 1, Irkutsk, p. 47. [In Russian]

Petrova NA (1988) Population cytogenetics of dipterous insects (on the example of blackflies and chironomids). In: Population Ecology, Part 1. Abstracts of the All-Union Meeting (4–6 October 1988, Novosibirsk), Moscow, pp. 138–140. [In Russian]

Petrova NA (1988) Results and prospects of the karyological study of chironomids. In: Xth International Symposium on Chironomidae, Debrecen, Hungary, July 25–28, 1988. Abstract Volume. Debrecen, p. 74.

Petrova NA (1988) Über die Möglichkeit der karyotypischer Artbestmmung macromorphologisch unbestimmbarer Chironomidenarten. In: XII. Internationales Symposium über Entomofaunistik in Mitteleuropa. Kiew, 25–30 September 1988. Kurzfassungen der Vorträge, Kiev, p. 128.

Petrova NA, Il’inskaya NB (1988) The model species *Chironomus plumosus* L. in the light of revised karyological data. In: Proceedings of the 5th All-Union Meeting ‘Species and Its Productivity in the Range’ (Tbilisi, 10–12 November 1988), Vilnius, pp. 261–263. [In Russian]

1989

Balushkina EA, Petrova NA (1989) Functioning of populations of chironomids in hypersalt lakes of the Crimea. In: The Investigations of the Water Ecosystem [sic!]. Trudy Zoologicheskogo Instituta AN SSSR (Proceedings of the Zoological Institute of the Academy of Sciences of the USSR), 205: 129–139. [In Russian]

Michailova P, Petrova NA (1989) Microevolution differentiation of *Pseudodiamesa* gr. *branickii* Nowicki (Diptera, Chironomidae). Tsitologiya 31(7): 824–828. [In Russian with English summary]

Petrova NA (1989) Characteristics of the karyotypes of midges (Diptera, Chironomidae) of the world fauna. I. Subfamilies Telmatogetoninae, Podonominae, Tanypodinae, Diamesinae, Prodiamesinae and Orthocladiinae. Entomologicheskoe Obozrenie 68(1): 107–120. [In Russian; English translation: Entomological Review 68(4): 68–85].

Petrova NA (1989) Chironomids of the subfamily Tanypodinae. II. Chromosome numbers of three species of Tanypodinae (Diptera, Chironomidae). In: Drjanovska OA (Ed.) Fourth National Conference on Cytogenetics with International Participation, 2–6 October, 1989, Vrantza, Bulgaria, pp. 192–194. [In Russian]

Petrova NA (1989) Results and prospects of the karyological study of chironomids. In: Dévai G (Ed.) Advances in Chironomidology: Proceedings of the Xth International Symposium on Chironomidae, Debrecen, 25–28 July, 1988. Pt. 1. Systematics, Molecular Biology, Cytology, Population Genetics, Zoogeography and Phenology. (Acta Biologica Debrecina. Supplementum Oecologica Hungarica, 2). Debrecen, pp. 295–304.

Petrova NA, Michailova PV (1989) Chromosome polymorphism of natural populations of *Endochironomus albipennis* Meig. (Diptera, Chironomidae). Tsitologiya 31(10): 1200–1205. [In Russian with English summary]

Petrova NA, Shobanov NA (1989) X International Symposium on the Chironomidae. Entomologicheskoe Obozrenie, 68(2): 447–449. [In Russian]

1990

Erbaeva EA, Zelentsov NA, Petrova NA, Narantuiaa G (1992) Morpho-karyological description of *Acricotopus lucens* Zett. (Chironomidae, Diptera) from the basin of Lake Khubsugul. In: Ganzorig S, Udval L (Eds) Natural Conditions and Resources of Some Regions of the Mongolian People’s Republic. Abstracts the conference, Ulaanbaatar, 1990, pp. 75–76. [In Russian]

Petrova NA (1990) Changes in the frequencies of chromosome structural rearrangements in chironomids from the Chernobyl zone from 1987 to 1988. In: Abstracts of the International Symposium on Chernobyl, 10–18.VI.1990. Ukraine, Zelenyi Mys, pp. 18–20. [In Russian]

Petrova N (1990) Characteristics of chironomid karyotypes (Diptera, Chironomidae) of the world fauna II. Subfamily Chironominae. Entomologicheskoe Obozrenie 69(1): 193–214. [In Russian]

Petrova NA (1990) Chromosomal polymorphism in *Chironomus balatonicus* of Chernobyl. In: Országh I (Ed.) Second International Congress of Dipterology, August 27 – September 1, 1990. Abstract Volume. Bratislava, p. 178. [In Russian]

Petrova NA, Il’inskaya NB (1990) Revision of populations of chironomids of plumosus group based on the analysis of fixed and fluctuating inversions. In: Khmeleva NN (Ed.) Species in Area: Biology, Ecology and Productivity of Water Invertebrates. Minsk: Navuka i Tekhnika, pp. 69–74. [In Russian]

1991

Michailova P, Petrova N (1991) Chromosome polymorphism in geographically isolated populations of *Chironomus plumosus* L. (Chironomidae, Diptera). Cytobios 67: 161–175.

Petrova NA (1991) Chromosomal rearrangements in three species of chironomids (Diptera, Chironomidae) from the Chernobyl zone. Genetika 27(5): 836–848. [In Russian with English summary]

Petrova NA (1991) Transformations of karyotype in evolution of Chironomidae and Simuliidae (Diptera). Comparative analysis. In: Abstracts of 11th International Symposium on Chironomidae, Amsterdam, 1991, p. 94.

Petrova NA, Michailova PV (1991) Inversion polymorphism of *Chironomus plumosus* (Diptera, Chironomidae) of Central and Eastern Europe. In: Vásárhelyi T, Zombori L (Eds) 4th European Congress of Entomology. XIII. Internationale Symposium für die Entomofaunistik Mitteleuropas. Abstract Volume, Gödöllö, p. 175.

1992

Il’inskaya NB, Petrova NA (1992) One more time about the standard karyotype of *Chironomus plumosus* L. and cytodiagnostics of its sibling species (Diptera, Chironomidae). Zoologicheskii Zhurnal 71(12): 76–86. [In Russian; English translation: On the typical karyotype of *Chironomus plumosus* and cytodiagnostic of “plumosus–group” species (Diptera, Chironomidae). Entomological Review (1993) 72(4): 135–147.]

Kachvoryan EA, Chubareva LA, Petrova NA (1992) [as “1991”] Comparative karyological analysis of two species of blackflies of the genus *Tetisimulium* Rubz. (Simuliidae, Diptera). In: Richter VA, Zlobin VV (Eds) Advantages of Entomology in USSR. Diptera: Systematics, Ecology, Medical and Veterinary Importance. St. Petersburg: Zoological Institute, Russian Academy of Sciences, pp. 145–148. [In Russian]

Petrova NA (1992) [as “1991”] Chromosomal aberrations in natural populations of chironomids from water bodies of Chernobyl. In: Richter VA, Zlobin VV (Eds) Advantages of Entomology in USSR. Diptera: Systematics, Ecology, Medical and Veterinary Importance. St. Petersburg: Zoological Institute, Russian Academy of Sciences, pp. 12–15. [In Russian]

Petrova N (1992) Polytene chromosomes of chironomids and simuliids and their role for studying the systematics and evolution of these groups. Abstract of Doctor of Biological Sciences Dissertation. St. Petersburg: Zoological Institute, Russian Academy of Sciences, 50 pp. [In Russian]

Petrova N (1992) Polytene chromosomes of chironomids and simuliids and their role for studying the systematics and evolution of these groups. Doctor of Biological Sciences Dissertation. St. Petersburg: Zoological Institute, Russian Academy of Sciences, 411 pp. and 154 pp. (Supplement). [In Russian]

Petrova NA, Michailova PV, Maximova FL, Il’inskaya NB (1992) The standard karyotype of *Chironomus plumosus* L. (Diptera, Chironomidae). Cytobios 70: 185–189.

Zelentsov NA, Petrova NA, Erbaeva EA (1992) Karyotype and morphology of *Acricotopus lucens* Zett. (Diptera, Chironomidae) from Mongolia. Entomologicheskoe Obozrenie 71(2): 295–301. [In Russian; English translation: Entomological Review (1993) 72(3): 28–34.]

1993

Il’inskaya N, Petrova N (1993) Cytodiagnostic [sic!] for larvae of the *Chironomus plumosus* group. Chironomus 5: 8.

Kiknadze II, Shobanov NA, Petrova NA, Belyanina SI (1993) Evolutionary aspects of the study of *Chironomus* species of the *plumosus* group (Chironomidae, Diptera). In: Proceedings of VI Meeting on the Project “Species and its Productivity in the Distribution Area”. UNESCO programme “Man and Biosphere” (St. Petersburg, 23–26 November 1993), St. Petersburg: Gidrometeoizdat, pp. 216–218. [In Russian]

Petrova NA (1993) Chromosomal polymorphism and microevolution of the Chironomidae and Simuliidae. In: Chubareva LA, Kuznetsova VG (Eds) Karyosystematics of Invertebrate Animals, II. St. Petersburg: Zoological Institute of the Russian Academy of Sciences, pp. 46–49. [In Russian with English summary]

Petrova NA (1993) Evolution of karyotypes in the Diptera with reference to Chironomidae and Simuliidae. In: Evolution 93: Fourth Congress of the European Society for Evolutionary Biology, Montpellier (France), August 22–28, 1993. Université Montpellier II, Sciences et techniques du Languedoc, p. 348.

Petrova NA (1993) Similarities and differences in the chromosome systems of the families Chironomidae and Simuliidae from the order Diptera. Chironomus 5: 8–9.

Zhirov SV, Petrova NA (1993) Karyotypical features of *Chironomus nuditarsis* Str. (Diptera) found in Russia for the first time. In: Chubareva LA, Kuznetsova VG (Eds) Karyosystematics of Invertebrate Animals, II. St. Petersburg: Zoological Institute of the Russian Academy of Sciences, pp. 50–51. [In Russian with English summary]

1994

Michailova P, Petrova N (1994) Cytogenetic characteristics of *Chironomus balatonicus* Devai, Wülker, Scholl (Diptera, Chironomidae) from the Chernobyl region. Cytobios 79: 15–29.

Petrova NA, Ivanchenko OV, Kerkis IE (1994) Cytogenetic structure of natural populations of *Chironomus balatonicus*. Tsitologiya 36(5): 469–478. [In Russian with English summary]

Petrova NA, Michailova P (1994) Genomic, chromosomal, chromatid and chromonemal abnormalities in *Chironomus balatonicus* from the Chernobyl zone. In: Proceedings of the 1st Congress of NI Vavilov Society of Geneticists and Breeders (VOGIS) (Saratov, 20–25 December 1994). Genetika 30, Supplement: 120–121. [In Russian]

1995

Michailova P, Petrova N, Ramella L, Regoli F, Sella G (1995) Genotoxic effects on the polytene chromosomes of a population of *Chironomus riparius* Meigen 1804 from a heavy metal polluted station. In: IV Congresso Nazionale della Società italiana di mutagenesi ambientale (S.I.M.A.), San Miniato, 16–19 Septembre 1995. Riassunti. Centro Studi “I Cappuccini” San Minuato Alto della Cassa di Risparmio di S. Minuato S. p. A., p. 46.

Michailova P, Petrova N, Ramella L, Sella G, Todorova D (1995) Effects of pollution on the polytene chromosomes of *Chironomus riparius* Meigen 1804 (Chironomidae, Diptera) from a river Po station. In: Abstracts of the 5th Congress of European Society for Evolutionary Biology, Edinburg, 4–8 September 1995, p. 232.

Petrova NA, Chubareva LA, Kachvoryan EA (1995) A new species of black flies, *Metacnephia paraskevae* sp. n. (Diptera, Simuliidae), from the East Pamir (Tajikistan). Entomologicheskoe Obozrenie 74(4): 899–901. [In Russian; English translation: Entomological Review (1996) 75(9): 227–231.]

Sella G, Michailova P, Petrova N, Ramella L, Todorova D (1995) Karyotype rearrangements in a population of *Chironomus riparius* Meigen 1804 from stressful environmental conditions. In: 6th Congresso Unione Zoologica Italiana (Reggio Calabria, 2–7 October 1995). Abstracts, pp. 192–193.

Shobanov NA, Petrova NA (1995) Karyotype peculiarities in *Chironomus saxatilis* Wülker et al., 1981 (Diptera, Chironomidae) from the Siberian Arctic region and a possible origin of neocentromeres [in chromosome AE (*pseudothummi* cytocomplex)]. Tsitologiya 37(7): 586–592. [In Russian with English summary]

1996

Chubareva LA, Petrova NA (1996) Karyotypic features and status of the superspecies taxa of the Palearctic blackfly subfamily Prosimuliinae (Diptera, Simuliidae). In: Gokhman VE, Kuznetsova VG (Eds) Karyosystematics of the Invertebrate Animals, III. Volume of Scientific Papers. Moscow, pp. 73–75. [In Russian with English summary].

Chubareva LA, Petrova NA, Kachvoryan EA (1996) Morpho-karyotypical features of four species of blackflies (Diptera: Simuliidae). Parazitologiya 30(1): 3–12. [In Russian with English summary]

Il’inskaya NB, Petrova NA (1996) Regularities of appearance of inversion polymorphism in the centre and at borders of range of *Chironomus plumosus*. In: Shobanov NA, Zinchenko TD (Eds) Ecology, Evolution and Systematics of Chironomidae. Togliatti, Borok, pp. 8–17. [In Russian]

Kachvoryan EA, Chubareva LA, Petrova NA, Mirumyan LS (1996) Frequency changes of B chromosomes in synanthropic species of bloodsucking blackflies (Diptera, Simuliidae). Genetika 32(5): 637–640. [In Russian; English translation: Russian Journal of Genetics 32(5): 554–557.]

Kerkis IE, Gordadze PR, Petrova NA, Chubareva LA (1996) One more time about the karyotype of *Prodiamesa olivacea* (Prodiamesinae, Chironomidae, Diptera). Tsitologiya, 38(3): 384–389. [In Russian with English summary]

Michailova P, Petrova N (1996) Chromosomal aberrations in irradiated natural population (Chernobyl) of *Chironomus balatonicus* (Chironomidae, Diptera). In: 5th International Congress of Systematic and Evolutionary Biology (August 17–24, 1996, Budapest). Abstracts, p. 318.

Michailova P, Petrova N, Ramella L, Sella G, Todorova J, Zelano V (1996) Cytogenetic characteristics of a population of *Chironomus riparius* Meigen 1804 (Diptera, Chironomidae) from a polluted Po river station. Genetica 98: 161–178. https://doi.org/10.1007/BF00121364

Michailova P, Petrova N, Sella G, Ramella L, Regoli F, Zelano V (1996) Genome mobilization in two populations of *Chironomus riparius* Meigen (Diptera, Chironomidae) from polluted sediments. In: XX International Congress of Entomology (Firenze, Italy, August 25–31, 1996). Proceedings, p. 257.

Petrova NA, Chubareva LA, Kachvoryan EA (1996) Structural changes in the karyotype of Palearctic blackflies during evolution of the family (Diptera, Simuliidae). In: Gokhman VE, Kuznetsova VG (Eds) Karyosystematics of the Invertebrate Animals, III. Volume of Scientific Papers. Moscow, pp. 59–61. [In Russian, English summary].

Petrova NA, Il’inskaya NB (1996) Features of inversion polymorphism in the natural populations of *Camptochironomus tentans* Fabricius from North-western part of Russia (Dipetra, Chironomidae). In: Gokhman VE, Kuznetsova VG (Eds) Karyosystematics of the Invertebrate Animals, III. Volume of Scientific Papers. Moscow, pp. 56–58. [In Russian, English summary].

Petrova NA, Il’inskaya NB, Kaidanov LZ (1996) Adaptiveness of inversion polymorphism in Chironomus plumosus (Diptera, Chironomidae): spatial distribution of inversions over species range. Genetika 32(12): 1629–1642. [In Russian; English translation: Russian Journal of Genetics 32(12): 1417–1430.]

Petrova NA, Michailova PV (1996) Three-year cytological research of *Chironomus balatonicus* from Chernobyl zone (1987–1989). In: Shobanov NA, Zinchenko TD (Eds) Ecology, Evolution and Systematics of Chironomidae. Togliatti, Borok, pp. 18–23. [In Russian]

Petrova N, Michailova P (1996) Cytogenetic monitoring of *Chironomus balatonicus* (Diptera, Chironomidae) from the Chernobyl region. International Journal of Dipterological Research 7(2): 79–86.

Petrova NA, Shobanov NA (1996) X conference on the Chironomidae. Entomologicheskoe Obozrenie 75(2): 478–479. [In Russian; English translation: Entomological Review (1996) 76(9): 1182–1183.]

1997

Bovero S, Michailova P, Petrova N, Ramella L, Regoli F, Sella G, Todorova D (1997) Genotoxic effects of chromium on IV chromosome of *Chironomus riparius*. In: Camatini M (Ed.) Abstracts of the International Symposium on Integrated Ecotoxicology “From Molecules/Organisms to Ecosystems”, Milano, June 29 – July 1, 1997, Ricerca Scientifica ed Educazione Permanente, Supplemento 109, p. 33.

Il’inskaya NB, Petrova NA (1997) Karyotype and inversion polymorphism of natural populations of *Camptochironomus tentans* from the North-Western region of Russia (Diptera, Chironomidae). Tsitologiya 39(9): 848–856. [In Russian with English summary]

Michailova P, Petrova N, Bovero S, Cavicchioli O, Ramella L, Sella G, Regoli F (1997) Correlation between chromium pollution and chromosomes rearrangements in natural population and a lab strain of *Chironomus riparius*. In: Atti Associazione Genetica Italiana XLIII, Orvieto (TR), 23–26 Settembre 1997, pp. 135–136.

Michailova P, Petrova N, Sella G, Ramella L, Bovero S (1997) Structure-functional alterations of chromosome G of *Chironomus riparius* (Diptera, Chironomidae) from a heavy metal polluted Piedmont station. In: 13th International Symposium on Chironomidae, Freiburg, September 5–9, 1997. Abstracts, p. 80.

Michailova P, Petrova N, Sella G, Ramella L, Todorova D, Bovero S (1997) The effect of some heavy metals on the polytene chromosomes of *Chironomus riparius* Mg. (Chironomidae, Diptera). In: Abstracts of VIII International “Balbiani Ring” Workshop, Falsterbo/Sweden, Aug. 30 – Sep. 3, 1997, pp. 26–27.

Michailova P, Petrova N, Sella G, Ramella L, Todorova D, Bovero S (1997) The effect of some heavy metals on the polytene chromosomes of *Chironomus riparius* Mg. (Chironomidae, Diptera). Hereditas 127: 274. <https://doi.org/10.1016/S0269-7491(98)00085-2>

Petrova NA, Chubareva LA (1997) Spatial and temporal regularities of the B-chromosomes appearance in chironomid midges and black flies populations. In: Nartshuk EP (Ed.) Diptera (Insecta) in Ecosystems [(21–25 April 1997, St. Petersburg). VI All-Russian Dipterological Symposium Dedicated to 100-years Anniversary of AA Stackelberg], St. Petersburg: Zoological Institute of Russian Academy of Sciences, pp. 94–95. [In Russian]

Sella G, Ramella L, Michailova P, Petrova N, Regoli F, Zelano V (1997) Effets genotoxiques des sediments pollues chez deux populations de *Chironomus riparius* Meigen 1804. In: Montunelle B (Ed.) Sédiments et gestion des milieu aquatiques. Actes du 4ème Congrès du GRAPE (Groupe Rhone Alpin Pollution Ecosystems), Lyon, France, 21–22 novembre 1995, pp. 98–101.

Shumakov EM, Petrova NA (1997) Results of the activity of the Specialized Council for dissertations defence for the degree of Doctor of Biological Sciences at the Zoological Institute of the Russian Academy of Sciences in the specialties ‘Entomology’ and ‘Parasitology’ (St. Petersburg). Entomologicheskoe Obozrenie 76(2): 485–490. [In Russian]

1998

Bovero S, Sella G, Michailova P, Petrova N, Hankeln T, Schmidt ER, Brunetti S, Ramella L (1998) Chromosome alterations and destabilization of repetitive DNA in a natural population *Chironomus riparius* from Piemont. In: Abstracts of the 13th International Chromosome Conference, Sept. 8–12, 1998, Acona, Italy. Cytogenetics and Cell Genetics 81: 125.

Chubareva LA, Petrova NA (1998) Evolution of karyotype in black flies (Diptera, Simuliidae) of the Palaearctic. In: Problems of Entomology in Russia. Collection of Scientific Papers of the XI Congress of the Russian Entomological Society (23–26 September 1997, St. Petersburg). Volume 2, St. Petersburg, pp. 203–204. [In Russian]

Kachvoryan EA, Petrova NA, Chubareva LA, Mirumyan LS (1998) Microevolutionary processes in populations of synanthropic species of black flies. In: Issues of Nature Conservation. Abstracts of Reports from Scientific Conference of 1998. Yerevan, pp. 66–67. [In Russian] <https://doi.org/10.1097/00007890-199810270-00097>

Michailova P, Petrova N, Sella G, Ramella L, Bovero S (1998) Structural-functional rearrangements in chromosome G in *Chironomus riparius* (Diptera, Chironomidae) collected from a heavy metal-polluted area near Turin, Italy. Environmental Pollution 103: 127–134. <https://doi.org/10.1016/S0269-7491(98)00085-2>

Petrova NA, Chubareva LA (1998) Results and perspectives of karyological studies of blackflies (Diptera, Simuliidae) of the Palaearctic. In: The VIth European Congress of Entomology, České Budějovice, Czech Republic, August 23–29, 1998. Book of Abstracts (Proceedings of the VIth European Congress of Entomology), pp. 422–423.

Petrova NA, Michailova P, Sella G, Ramella L, Zelano V, Regoli F (1998) Cytogenetic effect in populations of *Chironomus riparius* Mg. (Diptera, Chironomidae) inhabiting water bodies polluted with heavy metals. In: Problems of Entomology in Russia. Collection of Scientific Papers of the XI Congress of the Russian Entomological Society (23–26 September 1997, St. Petersburg). Volume 2, St. Petersburg, pp. 72–73. [In Russian]

1999

Bovero S, Hankeln T, Michailova P, Petrova N, Schmidt ER, Sella G (1999) Non random breaks in polytene chromosomes of larvae of *Chironomus riparius* (Diptera) reared on heavy metal polluted sediments. In: Haas OA (Coord) Abstracts of the Second European Cytogenetics Conference, July 3–6, 1999, Vienna, Austria. Cytogenetics and Cell Genetics 85(1–2) (Appendum): 788.

Bovero S, Michailova P, Petrova N, Ramella L, Sella G (1999) Spontaneous chromosomal alterations in two populations of *Chironomus riparius* in the surroundings of Turin. In: Seventh Congress of the European Society for Evolututionary Biology, Barcelona, Spain, 23–28 August, 1999, p. 271.

Chubareva LA, Petrova NA (1999) Karyotypic features and rank of supraspecific taxa in Palaearctic blackflies of the subfamily Prosimuliinae Enderlein (Diptera, Simuliidae). Entomologicheskoe Obozrenie 78(1): 189–195. [In Russian; English translation: Entomological Review 79(1): 50–57.]

Il’inskaya NB, Petrova NA, Matena I (1999) The relationship between the level of inversion polymorphism and the type of water body, the season, and the year of observation in *Chironomus plumosus* L. (Diptera, Chironomidae). Genetika 35(8): 1061–1070. [In Russian; English translation: Russian Journal of Genetics 35(8): 908–917.]

Michailova P, Petrova N, Bovero S, Hankeln T, Sella G, Ramella L, Schmidt E (1999) Chromosome rearrangements, heterochromatin and repetitive DNA elements in *Chironomus thummi* (Diptera, Chironomidae) from heavy metal polluted stations. In: IXth International Balbiani Ring Workshop, Sept. 12th–16th, Port Washington, Wisconsin, USA, p. 20.

Petrova NA, Chubareva LA (1999) Karyotype of prodiamesins [sic!] (Diptera, Chironomidae, Prodiamesinae) from the Bratsk Reservoir. Tsitologiya 41(1): 101–103. [In Russian with English summary]

Petrova NA, Chubareva LA, Kachvoryan E (1999) Chromosomal polymorphism in *Chironomus riparius* Meigen (Diptera, Chironomidae) from a southern peripheral population (the Armenian Upland). Tsitologiya 41(12): 1032–1037. [In Russian with English summary]

Petrova NA, Kupriyanova LA (1999) Paraskeva Michailova. “Eukaryotic chromosome – structure and function. Atlas”. Tsitologiya 41(10): 914–915. [Review of the book; in Russian]

2000

Cavichioli O, Giolitto R, Michailova P, Petrova N, Sella G (2000) Increased frequency of chromosomal rearrangements in *Chironomus riparius* polytene chromosomes from polluted sediments. In: Atti del X congresso nazionale della Società Italiana di Ecologia, Pisa, 14–16 settembre 2000. [not seen; page number(s) not identified]

Chubareva LA, Petrova NA (2000) Ecology and B-chromosomes in blackflies and chironomids (Diptera). In: Hunter FF (Ed.) Black Flies in the New Millennium: an International Meeting of Black Fly Workers, 17–21 June 2000. Brock University, St. Catharines, Ontario, Canada, p. 37.

Chubareva LA, Petrova NA (2000) Karyotypes of 17 species of blackflies (Diptera, Simuliidae) of the Murmansk Region. In: Ieshko EP, Nemova IN, Krutov VI, Kuznetsov OL (Eds) Conservation of Biological Diversity in Fennoscandia: Abstracts, presented to the International Conference (March 30 – April 2, 2000, Petrozavodsk). Petrozavodsk, 2000, pp. 104, 121. [In Russian and English]

Michailova P, Petrova N, Bovero S, Cavicchioli O, Ramella L, Sella G (2000) Effect of environmental pollution on the chromosomal variability of *Chironomus riparius* Meigen 1804 (Diptera, Chironomidae) larvae from two Piedmont stations. Genetica 108: 171–180. https://doi.org/10.1023/A:1004172019131

Michailova P, Petrova N, Bovero S, Hankeln T, Sella G, Ramella L, Schmidt E (2000) Structural and functional rearrangements in polytene chromosomes of chironomids (Diptera) as biomarkers for heavy metals pollutions in aquatic ecosystems. In: International Conference on Heavy Metals in the Environment, 6–10 August, 2000, University of Michigan, Ann Arbor, Michigan, USA. School Public Health, Contribution 1014: 70–75.

Petrova NA, Chubareva LA, Barabanova LV, Rybakova MV, Fyodorova ID (2000) The karyotype of the North Karelia new chironomids species (Diptera, Chironomidae). In: Ieshko EP, Nemova IN, Krutov VI, Kuznetsov OL (Eds) Conservation of Biological Diversity in Fennoscandia: Abstracts, presented to the International Conference (March 30 – April 2, 2000, Petrozavodsk). Petrozavodsk, 2000, pp. 78, 143. [In Russian and English]

Petrova N, Michailova P, Bovero S (2000) Cytogenetic characteristics of *Chironomus nuditarsis* Str. (Chironomidae, Diptera) and its relationship with species from the plumosus group. In: Hoffrichter O (Ed.) Late 20th Century Research on Chironomidae. An Anthology from the 13th International Symposium on Chironomidae, Freiburg, 5–9 September 1997, Aachen: Shaker Verlag, pp. 201–208.

Petrova NA, Michailova PV, Sella G, Ramella L, Bovero S, Zelano V, Regoli F (2000) Structural-functional alterations of polytene chromosomes of *Chironomus riparius* from some heavy metal-polluted water bodies of Italy. Sibirskii Ekologicheskii Zhurnal (Siberian Ecological Journal) 4: 511–521. [In Russian with English summary]

2001

Michailova P, Ilkova J, Petrova N, White K (2001) Rearrangements in the salivary gland chromosomes of *Chironomus riparius* Mg. (Diptera, Chironomidae) following exposure to lead. Caryologia 54(4): 349–363. <https://doi.org/10.1080/00087114.2001.10589246>.

Michailova P, Petrova N, Sella G, Bovero S, Ramella L, Regoli F, Zelano V (2001) Genotoxic effects of chromium on polytene chromosomes of *Chironomus riparius* Meigen 1804 (Diptera, Chironomidae). Caryologia 54(1): 59–71. <https://doi.org/10.1080/00087114.2001.10589213>

Petrova NA (2001) BRs system in Simuliidae and Chironomidae. Comparative cytogenetic aspect. In: Michailova P, Grozeva S, Genova G, Konova O, Dobrev D, Ilkova J, Mancheva A (Eds) Abstracts of Xth International Balbiani Ring Workshop. International House of Scientists, Varna, Bulgaria, August 31 – September 4, p. 25.

Petrova NA, Klishko OK (2001) Atypical puffing of *Chironomus plumosus* (Diptera, Chironomidae) in natural population from Chita Region. Tsitologiya 43(2): 172–177. [In Russian with English summary]

Petrova N, Mikhailova P, Sella G, Ramella L, Bovero S, Zelano F, Schmidt E, Hankeln T (2001) Relationship between the appearance of chromosomal inversions in *Chironomus riparius* and the pollution of water body by heavy metals (Diptera, Chironomidae). In: Rozenberg GS, Zinchenko TD (Eds) Small Rivers: Current Ecological State, Actual Problems. Abstracts of International Scientific Conference, Russia, Togliatti, 23–27 April 2001. Togliatti: Institute of Ecology of Volga Basin of the Russian Academy of Sciences, p. 164. [In Russian]

Rakisheva AZh, Petrova NA, Michailova P (2001) Larval morphology and karyotypic characteristics of *Chironomus jonmartini* Lindeberg (Diptera, Chironomidae) from peripheral southern population (Mountain Kazakhstan). Entomologicheskoe Obozrenie 80(2): 512–517. [In Russian; English translation: Entomological Review 81(9): 1079–1085.]

2002

Makarchenko EA, Klishko OK, Chubareva LA, Petrova NA (2002) Little known species of the genus *Propsilocerus* (Diptera, Chironomidae, Orthocladiinae) from the Chita Region (morphology and karyotypes). In: Medvedev GS (Ed.) XII Congress of the Russian Entomological Society, St. Petersburg, 19–24 August 2002. Abstracts. St. Petersburg, p. 224. [In Russian]

Petrova N, Michailova P (2002) Cytogenetic characteristics of *Chironomus bernensis* Klötzli (Diptera, Chironomidae) from a heavy metal polluted station in Northern Italy. Annales Zoologici 52(2): 227–233.

Petrova N, Michailova P, Ilkova J (2002) Variability of the polytene chromosomes in *Chironomus riparius* (Diptera, Chironomidae) of two wild populations from Bulgaria and Russia. In: Medvedev GS (Ed.) XII Congress of the Russian Entomological Society, St. Petersburg, 19–24 August 2002. Abstracts. St. Petersburg, p. 279. [In Russian]

2003

Chubareva LA, Petrova NA (2003) Karyotypes of blackflies (Diptera, Simuliidae) of the world. Entomologicheskoe Obozrenie 82(1): 157–222. [In Russian; English translation: Entomological Review 83(2): 149–204.]

Chubareva LA, Petrova NA, Kachvoryan EA (2003) Morphological diversity of centromere regions in polytene chromosomes of blackflies (Diptera, Simuliidae). Tsitologiya 45(4): 368–376. [In Russian with English summary.]

Kachvoryan EA, Petrova NA, Chubareva LA (2003) The results of karyological study of black flies (Diptera, Simuliidae) in Armenia. Parazitologiya 37(2): 89–102. [In Russian with English summary]

Karageuzyan KG, Chubareva LA, Kachvoryan EA, Adler PH, Petrova NA, Kyureghyan TN, Harutyunova LD, Hovhannisyan VS, Simonyan MA (2003) Ecological conditions of Hrazdan river (Armenia). Report I. Vestnik IAELPS, 8(4): 30–34. [In Russian with English summary]

Mikhailova P, Petrova N, Sella G, Bovero S, White K, Ramella L (2003) Cytogenetic biomarkers Chironomus riparius (Diptera) as indicators of heavy metal pollution. In: XV International Symposium on Chironomidae, 12–14 August 2003, the University of Minnesota, Minneapolis. Abstracts, p. 11.

Petrova NA, Chubareva LA, Adler PN [sic!], Kachvoryan EA (2003) Cytogenetic features of blood-sucking blackfly *Wilhelmia paraequina* Puri (Diptera: Simuliidae) from Armenia. Genetika 39(1): 41–50. [In Russian; English translation: Russian Journal of Genetics 39(1): 32–40. https://doi.org/10.1023/A:1022014725940]

Petrova NA, Klishko OK (2003) Cytogenetic peculiarities of three *Chironomus* species of plumosus group (Diptera, Chironomidae) from Chita Province of Russia. In: Shobanov NA (Ed.) New Data in Chironomidology. Borok: Institute for Biology of Inner Waters of the Russian Academy of Sciences, pp. 64–73. [In Russian with English summary]

Petrova NA, Rakisheva AZh (2003) Karyotype and morphology of the larvae of *Chironomus anthracinus* Zett. (Diptera, Chironomidae) from Eastern Kazakhstan. Tsitologiya 45(4): 428–433. [In Russian with English summary]

Petrova NA, Zelentsov NI, Klishko OK, Chubareva LA (2003) First description of polytene chromosomes, larval morphology and biology of two species of the genus *Propsilocerus* (Diptera, Chironomidae, Orthocladiinae). Trudy Russkogo Entomologicheskogo Obshchestva (Proceedings of the Russian Entomological Society) 74: 33–50. [In Russian]

Vinogradova EB, Petrova NA (2003) Synanthropization in chironomids: *Chironomus riparius* Mg. (Diptera: Chironomidae) as an example. In: Annual scientific session summarizing research in 2002. Abstracts. 8–10 April 2003. St. Petersburg: Zoological Institute of the Academy of Sciences of the Russian Academy of Sciences, pp. 11–12. [In Russian].

Vinogradova EB, Petrova NA (2003) Synanthropization in chironomids: *Chironomus riparius* (Diptera, Chironomidae) as an example. Trudy Zoologicheskogo Instituta RAS (Proceedings of the Zoological Institute of Russian Academy of Sciences) 299: 187–196.

Vinokurova NV, Veremeichik YaV, Petrova NA (2003) Aberrations of polytene chromosomes [in] larvae [of] *Chironomus plumosu*s from the Lake Shkol’noe in Kaliningrad. In: Shobanov NA (Ed.) New Data in Chironomidology. Borok: Institute for Biology of Inner Waters of the Russian Academy of Sciences, pp. 54–59. [In Russian with English summary]

2004

Chubareva LA, Petrova NA (2004) Characteristic features of karyotypes in common species of blackflies (Diptera, Simuliidae) from the northwestern region of Russia. Zoologicheskii Zhurnal 83(11): 1341–1352. [In Russian; English translation: Karyotypes in common blackfly species (Diptera, Simuliidae) of the northwestern region of Russia. Entomological Review 84(8): 866–877.]

Chubareva L, Petrova N (2004) Current knowledge of the karyotypes of the world blackfly fauna (Diptera, Simuliidae). Deutsche Gesellschaft für allgemeine und angewandte Entomologie (DGaaE) Nachrichten 18(3): 102–103. [The identical paper was published in British Simuliid Group Bulletin 23: 23–24 (2005).]

Michailova P, Petrova N (2004) Natural hybridization in insects, Diptera (model group – family Chironomidae). In: Bakerdzhieva N, Michailova P (Eds) Evolution and Ecology – 2004. Seminar Dedicated to the 60th Anniversary of the Union of Scientists in Bulgaria. Proceedings. Sofia, pp. 9–19.

Petrova NA, Chubareva LA, Kachvoryan EA (2004) The karyotypical peculiarities and inversion polymorphism of *Prodiamesa olivacea* Mg. (Diptera, Chironomidae, Prodiamesinae) from Armenia. Vestnik IAELPS, 9(3): 26–29. [In Russian with English summary]

Petrova NA, Michailova P, Ilkova J (2004) Comparative cytogenetic variation of the salivary gland polytene chromosomes in *Chironomus riparius* Mg., 1804 (Diptera, Chironomidae) from two polluted biotopes of Bulgaria and Russia. Genetika 40(1): 49–58. [In Russian; English translation: Russian Journal of Genetics 40: 40–48. <https://doi.org/10.1023/B:RUGE.0000013447.90957.1a>]

Petrova NA, Michailova PV, Chubareva LA, Shobanov NA, Zelentsov NI (2004) The system of A.A. Chernovskii [1949] as a basis for the cytotaxonomy of the family Chironomidae. Euroasian Entomological Journal 3(4): 253–258. [In Russian with English summary]

Sella G, Bovero S, Ginepro M, Michailova P, Petrova N, Robotti CA, Zelano V (2004) Inherited and somatic cytogenetic variability in Palearctic populations of *Chironomus riparius* Meigen 1804 (Diptera, Chironomidae). Genome 47: 332–344. https://doi.org/10.1139/g03-128.

Vinogradova EB, Petrova NA (2004) First record of a synanthropic population of *Chironomus riparius* Meigen, 1804 (Diptera, Chironomidae) in dwelling house basements in St. Petersburg and some of its biological and karyological characteristics. Entomologicheskoe Obozrenie 83(2): 334–348. [In Russian; English translation: Entomological Review 84(7): 752–763.]

2005

Michailova P, Petrova N (2005) Comparative effect of heavy metals on the polytene chromosomes of Chironomidae, Diptera. In: Gruev B, Nikolova M, Donev A (Eds) Proceedings of the Balkan Scientific Conference of Biology in Plovdiv (Bulgaria) from 19th till 21st of May 2005, pp. 539–552.

Michailova P, Petrova N, Ilkova J, Bovero S, Brunetti S, White K, Sella G (2005) Acute concentrations of aqueous Cu result in numerous changes of structure and function of the polytene chromosomes of chironomid larvae. In: Atti del XV Congresso Nazionale della Società Italiana di Ecologia, 12–14 September, 2005, Torino, p. 132.

Michailova P, Petrova N, White K, Sella G (2005) Cytogenetic indicators of trace metal pollution in chironomids under chronic treatment. In: Atti del XV Congresso Nazionale della Società Italiana di Ecologia, 12–14 September, 2005, Torino, p. 133.

Petrova NA, Klishko OK (2005) Cytodiagnosis, inversion polymorphism, and B-chromosomes of three *Chironomus* sibling species of the group plumosus (Diptera, Chironomidae) from Eastern Siberia. Zoologicheskii Zhurnal 84(7): 838–849. [In Russian; English translation: Entomological Review 85(7): 729–740.]

Petrova NA, Vinokurova NV, Danilova MV (2005) Some karyotype features of Camptochironomus tentans population from a northwestern region of Russia. In: Materials of International Scientific Conference ‘Actual Problems of Ecology, Physiology, Biochemistry and Genetics of Animals’. Saransk: Mordovian Universtity Press, pp. 173–175. [In Russian]

2006

Chubareva LA, Petrova NA (2006) B chromosome polymorphism of blackflies (Diptera, Simuliidae) from the north-western region of Russia. Tsitologiya 48(3): 258–263. [In Russian with English summary]

Chubareva LA, Petrova NA (2006) Karyotaxonomy of blackflies (Diptera: Simuliidae). In: Kuznetsova VG (Ed.) Book of Abstracts / IV International Conference on Karyosystematics of the Invertebrates, 28–30 August 2006, St. Petersburg, Russia, p. 57. [In Russian]

Kuznetsova VG, Petrova NA, Kupriyanova LA, Golub NV, Khabazova NS, Korolyova YuI, Kaidanova TI, Grinchuk TM, Petrukhina TE, Kachvoryan EA, Grozeva S, Mikhailova P et al. (2006) [“2005”] Jubilaeum of Professor Lidia Arkhipovna Chubareva. Russian Entomological Journal 14(4): 347–349. [In Russian]

Michailova P, Ilkova J, Sella G, Petrova N, Selvaggi A (2006) Chromosome rearrangements, repetitive DNA clusters and transposable elements in *Chironomus riparius* Mg. (Diptera: Chironomidae) from anthropogenically polluted regions. In: Kuznetsova VG (Ed.) Book of Abstracts / IV International Conference on Karyosystematics of the Invertebrates, 28–30 August 2006, St. Petersburg, Russia, p. 38.

Michailova P, Ilkova J, Sella G, Zampicinini GP, Petrova N, White K (2006) Genome instability of *Chironomus riparius* Mg. and *Chironomus piger* Strenzke (Diptera, Chironomidae) from anthropogenic polluted regions. In: 16th International Chironomid Symposium, 25th–28th July 2006, Casa da Luz Museum, Funchal, Madeira, Portugal, p. 84.

Michailova P, Petrova N, Ilkova J, Bovero S, Brunetti S, White K, Sella G (2006) Genotoxic effect of copper on salivary gland polytene chromosomes of *Chironomus riparius* Meigen 1804 (Diptera, Chironomidae). Environmental Pollution 144: 647–654. https://doi.org/10.1016/j.envpol.2005.12.041

Petrova NA, Chubareva LA (2006) Karyotypes of Siberian and Far-Eastern blackflies (Diptera, Simuliidae). Euroasian Entomological Journal 5(2): 111–121. [In Russian with English summary]

Petrova NA, Chubareva LA, Reva MV (2006) Polymorphism of the hematophagous black fly *Boophthora erythrocephala* (Diptera, Simuliidae) natural populations. In: Medvedev SG (Ed.) [Materials of the] I Russian Conference on Bloodsucking Insects, St. Petersburg, October 24–27, 2006, St. Petersburg: Zoological Institute of the Russian Academy of Sciences, pp. 166–167. [In Russian]

Vinokurova NV, Danilova MV, Petrova NA (2006) Some karyotype features of *Camptochironomus tentans* from a northwest region of Russia (Kaliningrad). In: Kuznetsova VG (Ed.) Book of Abstracts / IV International Conference on Karyosystematics of the Invertebrates, 28–30 August 2006, St. Petersburg, Russia, p. 13. [In Russian]

Vinokurova NV, Petrova NA, Danilova MV (2006) Some karyotype features of *Camptochironomus tentans* population from a northwestern region of Russia, city of Kaliningrad. In: Alimov AF, Rosenberg GS (Eds) IX Congress of the Russain Hydrobiological Society (Togliatti, Russia, 18–22 September 2006), Volume 1, Togliatti: Institute of Ecology of Volga Basin of the Russian Academy of Sciences, p. 83. [In Russian]

2007

Chubareva LA, Petrova NA (2007) Karyological characters of blackflies (Diptera: Simuliidae). Comparative Cytogenetics 1(1): 89–94.

Chubareva LA, Petrova NA, Reva MV (2007) Karyotypic and morphological study of five species of the genus *Wilhelmia* Enderlein (Diptera, Simuliidae). Entomologicheskoe Obozrenie 86(6): 895–904. [In Russian; English translation: Entomological Review 87(9): 1290–1299.]

Ilkova J, Hankeln T, Schmidt ER, Michailova P, Petrova N, Sella G, White K (2007) Genome instability of *Chironomus riparius* Mg. and *Chironomus piger* Strenzke (Diptera, Chironomidae). Caryologia 60(4): 299–308. https://doi.org/[10.1080/00087114.2007.10797951](http://dx.doi.org/10.1080/00087114.2007.10797951)

Kachvoryan EA, Oganesyan VS, Petrova NA, Zelentsov NI (2007) The fauna of chironomids and blackflies (Diptera: Chironomidae, Simuliidae) and hydrochemical characteristics of the Hrazdan River (Armenia). Entomologicheskoe Obozrenie 86(1): 104–113. [In Russian; English translation: Entomological Review 87(1): 73–81. https://doi.org/10.1134/S0013873807010071]

Kuznetsova VG, Anokhin BA, Golub NV, Nechaeva GA, Petrova NA, Labina ES, Gavrilov IA (2007) Use of cytogenetic and molecular markers for studying the dynamics of gene pools in insects (Insecta) and coelenterates (Cnidaria). In: Dynamics of Gene Pools. Subprogram 2. Materials of the Conference Dedicated to the Memory of YuP Altukhov. Moscow, p. 25–28. [In Russian]

Kuznetsova VG, Kupriyanova LA, Lukhtanov VA, Petrova NA (2007) Followers and students of ME Lobashev at the Zoological Institute of Russian Academy of Sciences: personalities, scientific and pedagogical activity. In: Systemic Control of Genetic and Cytogenetic Processes. International School-Conference Dedicated to the 100th Anniversary of ME Lobashev, St. Petersburg, 10–13 November 2007. St. Petersburg, pp. 20–21. [In Russian]

Michailova P, Ilkova J, Petrova N, Selvaggi A, Zampicinini GP, Sella G (2007) The relationship between chromosome rearrangements and repetitive DNA clusters in *Chironomus riparius* Meigen (Diptera: Chironomidae) from anthropogenically polluted Palaearctic regions. Comparative Сytogenetics 1(1): 45–49.

Michailova P, Sella G, Petrova N (2007) Effect of heavy metals on the genome of model insect group (Diptera, Chironomidae). In: Evolution and Ecology – 2007. Proceedings. Sofia, pp. 36–43.

Petrova NA (2007) Karyological evidence for tandem chromosome fusion in *Stictochironomus* sp. (Chironomidae). In: Problems and Perspectives of General Entomology. Proceedings of XIII Congress of the Russian Entomological Society, Krasnodar, 9–15 September 2007. St. Petersburg, pp. 275–276. [In Russian]

Petrova NA, Chubareva LA, Reva MV (2007) Cytogenetic analysis of the noxious bloodsucker *Boophthora erythrocephala* (Diptera: Simuliidae) from different geographic zones. Tsitologiya 49(4): 329–339. [In Russian with English summary]

Petrova NA, Vinokurova NV, Danilova MV (2007) Chromosomal variation of populations of *Chironomus plumosus* Linnaeus (Diptera: Chironomidae) from lakes of Kaliningrad, Russia. Comparative Cytogenetics 1(1): 51–54.

Petrova NA, Vinokurova NV, Danilova MV, Maslova VV (2007) Seasonal variability of the karyotype structure of *Chironomus plumosus* (Diptera, Chironomidae) from a biotope of Kaliningrad. Tsitologiya 49(10): 901–905. [In Russian with English summary]

2008

Chubareva LA, Petrova NA (2008) Atlas of polytene chromosomes of 123 species of blackflies from the Palearctic. In: The 3rd International Simuliidae Symposium, including the 29th Meeting of the British Simuliid Group, the 7th European Simuliidae Symposium and EMCA Blackfly Working Group. Vilnius, September 9th–12th, 2008. Abstracts. Vilnius, Lithuania: Lithuanian State Science and Studies Foundation, p. 39.

Chubareva LA, Petrova NA (2008) Cytogenetic maps of polytene chromosomes and some morphological peculiarities of blackflies from Russia and adjacent territories (Diptera, Simuliidae). Atlas. St. Petersburg, Moscow: KMK Scientific Press, 135 pp., 218 plates. [In Russian]

Ilkova J, Michailova P, Petrova N, Sella G, Hankeln T, Schmidt E (2008) Repetitive DNA localization in two homosequential species of the genus *Chironomus* Kieffer (Diptera, Chironomidae) and their genome reaction to anthropogenic factors. Acta Zoologica Bulgarica, Supplement 2: 41–48.

Kachvoryan EA, Petrova NA, Harutyunova LD, Zelentsov NI, Zhirov SV, Hovhannisyan VS (2008) Biodiversity of invertebrate animals in relation to water quality in the Hrazdan River system of Armenia. In: International Conference ‘State-of-the-Art Biotechnology in Armenia & ISTC Contribution. Book of Abstracts. Tsakhadzor, Republic of Armenia, September 28 – October 02, 2008, pp. 55–56. [In English and Russian]

Petrova NA, Zhirov SV (2008) Cytogenetics of *Chironomus riparius* L. from the fishpond in Borok village (Diptera, Chironomidae, Diptera). Tsitologiya 50(6): 535–538. [In Russian with English summary]

Petrova NA, Zhirov SV (2008) Polytene chromosomes of salivary glands of chironomids (Diptera: Chironomidae) from the Wrangel Island (Russia). Comparative Cytogenetics 2(2): 127–130.

2009

Chubareva LA, Petrova NA (2009) Atlas of polytene chromosomes of 123 species of blackflies from the Palearctic. British Simuliid Group Bulletin 31 (Supplement): 28.

Chubareva LA, Petrova NA (2009) Karyotypes of rare and little-known species of blackflies (Diptera, Simuliidae). Acta Zoologica Lithuanica 19(3): 175–177. https://doi.org/10.2478/v10043-009-0022-7

Chubareva LA, Petrova NA (2009) Survey of polytene chromosomes of 124 species of blood-sucking midges (Diptera, Simuliidae). In: Congress of Geneticists and Breeders Dedicated to the 200th Anniversary of Charles Darwin’s Birth. V Congress of the Vavilov Society of Geneticists and Breeders. Part II. Moscow, 21–28 June 2009. Moscow: AK Timiryazev Russian Academy of Agricultural Sciences, p. 195. [In Russian]

Editorial Board of Comparative Cytogenetics [Petrova NA et al.] (2009) In memoriam: Doctor Egeny Kachvoryan (2.III.1942–6.XII.2008). Comparative Cytogenetics, 3(1): 79–81. <https://doi.org/10.3897/compcytogen.v3i1.11>

Petrova NA, Zhirov SV (2009) Inversion polymorphism in two chironomid species of the genera *Chironomus* and *Camptochironomus* (Diptera, Chironomidae, Chironomini) from different regions of Russia (central part and North-West). Vestnik Sankt-Peteburgskogo Gosudarstvennogo Universiteta, Series 3, Biologiya, 4: 29–39, 149–150, 154–155. [In Russian]

Sharton AYu, Petrova NA, Vinokurova NV, Danilova MV (2009) Chironomid larvae (Diptera, Chironomidae) as indicators of pollution of water bodies with heavy metals. In: Ecology, Evolution and Systematics of Animals. Materials of All-Russian Scientific-Practical Conference with International Participation, 17–19 November 2009, Ryazan: NP ‘Golos Gubernii’, pp. 157–158. [In Russian]

2010

Ilkova J, Michailova P, Petrova N, White K, Sella G, Hankeln T, Schmidt E (2010) Genome response of phylogenetically different species of the family Chironomidae (Diptera). In: International Conference on Karyosystematics of Invertebrates V. August 16–20 2010. Novosibirsk [Program and Materials], p. 52.

Michailova P, Petrova N, Sella G (2010) Genome response of model insect group (Chironomidae Diptera) to trace metal contaminants in the environment. In: Recent Advances in Mathematics and Computers in Business, Economics, Biology and Chemistry. Proceedings of the 11th WSEAS International Conference on Mathematics and Computers in Business and Economics (MCBE’10). “G. Enescu” University, Iasi, Romania, June 13–15, 2010, pp. 366–372.

Michailova P, Petrova N, Sella G, Bovero S, White K, Ramella L (2010) Cytogenetic biomarkers in *Chironomus riparius* Mg. (Diptera) as indicators of heavy metal pollution. In: Ferrington LC, Jr (Ed.) Proceedings of the XV International Symposium on Chironomidae. Saint Paul, Minnesota: University of Minnesota, pp. 235–242.

Petrova NA (2010) In memory of EA Kachvoryan (1942–2008). Entomologicheskoe Obozrenie 89(3): 696–700. [In Russian] <https://doi.org/10.1134/S0013873810080191>

Petrova N, Michailova P, Bovero S, Sella G (2010) Karyological and morphological studies of some chironomids species from Northern Italy (Diptera, Chironomidae). In: International Conference on Karyosystematics of Invertebrates V. August 16–20 2010. Novosibirsk [Program and Materials], p. 63.

Petrova NA, Zhirov SV (2010) Larvae morphology, karyotype structure, and inversion polymorphism in a chironomid from the Republic of South Africa (Diptera, Chironomidae). Vestnik VOGiS 14(1): 70–78. [In Russian with English summary]

Sharton AYu, Petrova NA, Vinokurova NV, Danilova MV, Zolotova SM (2010) Inversion polymorphism of *Glyptotendipes glaucus* Mg. (Diptera: Chironomidae) from the reservoirs of Kaliningrad. Genetika 46(7): 887–895. [In Russian; English translation: Russian Journal of Genetics 46(7): 786–793. <https://doi.org/10.1134/S1022795410070021>]

Zinchenko TD, Petrova NA (2010) In memory of Ezheni Ashotovna Kachvoryan (2.03.42 – 6.12.08). Samarskaya Luka: Problems of Regional and Global Ecology 19(3): 200–244. [In Russian]

2011

Michailova P, Sella G, Petrova N (2011) Chironomids (Diptera) and their salivary gland chromosomes as indicators of trace-metal genotoxicity. Italian Journal of Zoology 79(2): 218–230. <https://doi.org/10.1080/11250003.2011.622084>

Michailova P, Sella G, Petrova N (2011) Polytene chromosomes of Chironomidae (Diptera) as a bioassay of trace-metal-induced genome instability. In: 18th International Symposium on Chironomidae, Trondheim, Norway, 4–6 July 2011. Scientific Program and Abstracts, NTNU Museum of Natural History and Archaeology, p. 35.

Petrova NA, Vinokurova NV, Danilova MV, Sharton AYu (2011) Inversion polymorphism in the population of *Camptochironomus tentans* from Kaliningrad city. Tsitologiya 53(7): 580–585. [In Russian with English summary]

Petrova NA, Zhirov SV (2011) Cytogenetic comparison of chironomid midge *Glyptotendipes glaucus* (Meigen, 1818) (Diptera, Chironomidae) populations from northwest Russia and Ukraine (Chernobyl zone). Ekologicheskaya Genetika (Ecological Genetics) 9(2): 9–16. [In Russian with English summary] <https://doi.org/10.17816/ecogen1029-16>

Petrova NA, Zhirov SV, Zelentsov NI, Kachvoryan EA (2011) To the fauna of chironomids (Diptera, Chironomidae) from the Razdan River valley (Armenia). Zoologicheskii Zhurnal 90(4): 445–451. [In Russian; English translation: On the fauna of Chironomidae (Diptera) of the Hrazdan Basin (Armenia). Entomological Review 91(3): 360–366. https://doi.org/10.1134/S0013873811030110]

2012

Michailova P, Sella G, Petrova N (2012) Polytene chromosomes of Chironomidae (Diptera) as a bioassay of trace-metal-induced genome instability. In: Ekrem T, Stur E, Aagaard K (Eds) Proceedings of the 18th International Symposium on Chironomidae. Fauna Norvegica 31: 227–234. <https://doi.org/10.5324/fn.v31i0.1355>

Petrova NA, Zhirov SV (2012) Karyosystematics of Chironomidae (Diptera) and borders of ranges. In: Belokobylskij SA (Ed.) XIV Congress of Russian Entomological Society. St. Petersburg, August 27 – September 1, 2012. Materials of the Congress. St. Petersburg, p. 341. [In Russian]

Petrova NA, Zhirov SV, Harutyunova KV, Harutyunova KV (2012) Morphological deformations of mouth parts in some species of the subfamilies Orthocladiinae and Diamesinae (Diptera, Chironomidae). Biological Journal of Armenia, 64(4): 48–52. [In Russian with English summary]

Petrova NA, Zhirov SV, Harutyunova MV, Harutyunova KV (2012) Cytotaxonomy and morphology of chironomid larvae (Diptera, Chironomidae) in Armenia. World Academy of Science, Engineering and Technology, 65: 513–517.

2013

Petrova NA (2013) Reorganization of polytene chromosomes of chironomid larvae (Diptera, Chironomidae) as a response to mutagenic pollution of environment (Chernobyl ecocatastrophe). St. Petersburg: Zoological Institute of Russian Academy of Sciences, 98 pp. [In Russian]

Petrova NA, Michailova PV, Bovero S, Sella G (2013) Karyotypes of four species of chironomids (Diptera, Chironomidae) from Northern Italy. Tsitologiya 55(6): 436–441. [In Russian; English translation: Cell and Tissue Biology 7(5): 465–471. <https://doi.org/10.1134/S1990519X13050088>]

Petrova NA, Zhirov SV (2013) Characteristics of the karyotypes of three subfamilies of chironomids (Diptera, Chironomidae: Tanypodinae, Diamesinae, Prodiamesinae) of the world fauna. Entomologicheskoe Obozrenie 92(3): 505–516. [In Russian; English translation: Entomological Review (2014) 94(2): 157–165. <https://doi.org/10.1134/S001387381402002X>]

Zhirov SV, Petrova NA (2013) The chironomid midge *Dicrotendipes* sp. afr. (Diptera, Chironomidae) from the Republic of South Africa. Zoologicheskii Zhurnal 92(4): 464–471. [In Russian; English translation: Entomological Review 93(6): 695–702. <https://doi.org/10.1134/S0013873813060031>]

2014

Karmokov MKh, Belyanina SI, Zhirov SV, Petrova NA (2014) Karyotype and morphology of a midge *Stictochironomus crassiforceps* (Kieffer) (Diptera, Chironomidae) in several parts of the Palaearctic Region. Entomologicheskoe Obozrenie 93(3–4): 555–563. [In Russian; English translation: Entomological Review 94(9): 1229–1238. https://doi.org/10.1134/S0013873814090048]

Petrova NA, Zhirov SV (2014) Karyology of chironomid larvae (Diptera, Chironomidae) of Kunashir Island (Kuril Ridge). In: VI Congress of Vavilov Society of Geneticists and Breeders (VOGiS) and Associated Genetic Symposia. Rostov-on-Don, 15–20 June 2014. Abstracts of Reports, pp. 16–17. [In Russian]

Petrova NA, Zhirov SV, Erbaeva EA (2014) Description of three species of chironomids (Diptera, Chironomidae) from Lake Khubsugul, Mongolia (morphological and karyological aspects). Euroasian Entomological Journal 13(5): 445–450. [In Russian with English summary].

Petrova NA, Zhirov SV, Harutyunova K, Harutyunova M (2014) On the possibility of spontaneous interspecific hybridization in the nature of representatives of sibling-species *Chironomus riparius* Kieffer and *Chironomus piger* Strenzke (Diptera, Chironomidae) from Armenia. Tsitologiya 56(2): 170–174. [In Russian with English summary]

2015

Lobkova LE, Orel OV, Zhirov SV, Petrova NA (2015) Chironomus (Chironomus) acidophilus Keyl, 1960 (Diptera, Chironomidae, Chironominae): biology, morphology, karyotype and habitat conditions in the caldera of the Uzon Volcano (Kamchatka, Kronotsky Nature Reserve). In: Lobkov EG (Ed.) Trudy Kronotskogo Gosudarstvennogo Prirodnogo Biosfernogo Zapovednika, 4: 92–119, 176. Petropavlovsk-Kamchatskii: Kamchatpress. [In Russian with English summary]

Michailova P, Petrova NA (2015) Bioindicator potential of cytogenetic variability in polytene chromosomes of chironomids (Diptera, Chironomidae) to assess environmental pollution. Tsitologiya i Genetika 49(4): 61–70. [In Russian; English translation: Cytology and Genetics, 49(4): 262–269. <https://doi.org/103103/S0095452715040064>]

Orel OV, Lobkova LE, Zhirov SV, Petrova NA (2015) A new record of *Chironomus* (*Chironomus*) *acidophilus* Keyl (Diptera, Chironomidae) from the Uzon volcanic caldera (Kronotsky Reserve, Kamchatka Peninsule, Russia), its karyotype, ecology and biology. Zootaxa 3981(2): 177–192. <https://doi.org/10.11646/zootaxa.3981.2.2>

Petrova NA, Cornette R, Shimura S, Gusev O, Pemba D, Kikawada T, Zhirov S, Okuda T (2015) Karyotypical characteristics of two allopatric African populations of anhydrobiotic *Polypedilum* Kieffer, 1912 (Diptera, Chironomidae) originating from Nigeria and Malawi. Comparative Cytogenetics 9(2): 173–188. <https://doi.org/10.3897/CompCytogen.v9i2.9104>

Petrova NA, Dzhaparidze LA, Zhirov SV (2015) On the need for revision of diploid chromosome numbers in some species of chironomids (Diptera, Chironomidae). In: Proceedings of the XX International Scientific and Practical Conference ‘Modern Concepts of Scientific Research’, Moscow, 27–28 November 2015. Eurasian Union of Scientists 11(20): 26–28. [In Russian]

Petrova NA, Zhirov SV (2015) The cytogenetic characteristic of some Palearctic populations of Holarctic midge *Glyptotendipes barbipes* Staeger (Diptera, Chironomidae). Tsitologiya 57(11): 831–837. [In Russian with English summary]

Zhirov SV, Petrova NA (2015) Karyotypes and larval morphology of three species of midges (Diptera, Chironomidae) from lakes in the southern part of Kunashir Island. Entomologicheskoe Obozrenie 95(3): 599–607. [In Russian; English translation: Entomological Review 95(7): 881–890. <https://doi.org/10.1134/S0013873815070064>]

2016

Petrova NA, Zhirov SV (2016) The role of salivary gland polytene chromosomes of chironomid (Diptera, Chironomidae) larvae in species identification and bioindication of environment pollution. In: X All-Russian Dipterolical [sic!] Symposium (with International Participation), Krasnodar, 23–28 August 2016. Materials. Krasnodar: Kuban State University, pp. 267–274. [In Russian with English summary]

Zhirov SV, Petrova NA, Krasheninnikov AB (2016) *Chironomus albimaculatus* and *Chironomus tuvanicus* (Diptera, Chironomidae) from the Novaja Zemlja [sic!] islands. In: Durnova NA (Ed.) Materials. VI International Conference on the Karyosystematics of the Invertebrates, 27–30 August, 2016, Saratov, Russia. Saratov, p. 21. [In Russian]

2017

Cornette R, Yamamoto N, Yamamoto M, Kobayashi T, Petrova NA, Gusev O, Shimura S, Kikawada T, Pemba D, Okuda T (2017) A new anhydrobiotic midge from Malawi, *Polypedilum pembai* sp. n. (Diptera: Chironomidae), closely related to the desiccation tolerant midge, *Polypedilum vanderplanki* Hinton. Systematic Entomology 42: 814–825. <https://doi.org/10.1111/syen.12248>

Petrova NA, Zhirov SV (2017) Karyotype characteristics of *Chironomus fraternus* Wülker and *Ch. beljaninae* Wülker (Diptera, Chironomidae) from Northern Russia. Entomologicheskoe Obozrenie 96(3): 429–435. [In Russian; English translation: Entomological Review 97(6): 730–734. <https://doi.org/10.1134/S0013873817060033>]

Zhirov SV, Petrova NA, Przhiboro AA (2017) First description of the karyotype of polytene chromosomes of «Orthocladiinae acuticauda» or «Orthocladiine aus Fluβsand» (Diptera: Chironomidae: Orthocladiinae). In: Barkalov AV (Ed.) XV Congress of Russian Entomological Society. Novosibirsk, July 31 – August 7, 2017. Materials of the Congress. Novosibirsk, pp. 188–189. [In Russian]

2018

Мichailova P, Kuznetsova V, Grozeva S, Ilkova J, Petrova N (2018) In Memoriam: Professor Iya Kiknadze (1930–2017). Comparative Cytogenetics 12(1): 141–144. <https://doi.org/10.3897/CompCytogen.v12i1.24550>

Przhiboro A, Saidov A, Petrova N (2018) Firuz Akhrorov (1937–2012) and his contributions to the study of Chironomidae in Tajikistan. In: Lencioni V, Cranston PS, Makarchenko EA (Eds) Recent advances in the study of Chironomidae: An overview. Journal of Limnology 77(s1): 10–14. <https://doi.org/10.4081/jlimnol.2018.1800>

2019

Petrova NA (2019) Cytogenetic features of chironomids. In: Proceedings of the VII Congress of NI Vavilov Society of Geneticists and Breeders (VOGiS) Dedicated to the 100th Anniversary of the Department of Genetics of St. Petersburg State University, and Associated Symposia, St. Petersburg, 18–22 June 2019. [In Russian; not seen; page number(s) not identified]

Petrova NA, Zhirov SV, Krasheninnikov AB (2019) Larval morphology and karyotype structure of *Chironomus* sp. (Diptera, Chironomidae) from the Novaya Zemlya Archipelago (Russia). Entomologicheskoe Obozrenie 98(4): 761–771. <https://doi.org/10.1134/S0367144519040099> [In Russian; English translation: Entomological Review 99(8): 1183-1191. <https://doi.org/10.1134/S0013873819080128>]

Kuznetsova V, Golub N, Petrova N, Lukhtanov V, Anokhin B, Khabazova N, Shapoval N, Kupriyanova L, Gavrilov-Zimin I (2019) In Memoriam: Dr. Sergey V. Zhirov (1966–2017). Comparative Cytogenetics 13(3): 321–324. <https://doi.org/10.3897/CompCytogen.v13i3.47366>

2020

Markiyanova MF, Petrova NA (2020) Chromosomal variability in *Chironomus plumosus* (Linnaeus, 1758) (Diptera, Chironomidae) from a coastal lagoon of the Baltic Sea (Curonian Lagoon). Zoologicheskii Zhurnal 99(9): 1002–1013. <https://doi.org/10.31857/S0044513420090135> [In Russian; English translation: Entomological Review 100(7): 969–981. <https://doi.org/10.1134/S0013873820070027>]

Petrova NA (2020) Chromosome evolution in Chironomidae (Diptera, Chironomidae). In: Ovchinnikova OG, Shamshev IV (Eds) XI All-Russian Dipterological Symposium (with international participation), Voronezh, 24–29 August 2020. Materials. St. Petersburg: Russian Entomological Society: LEMA Publ., pp. 188–192. [In Russian with English summary] <https://doi.org/10.47640/978-5-00105-586-0_2020_188>

2021

Petrova NA (2021) Ninel Alekseevna Petrova. In: Brodskaya NK, Dunaeva YuA, Przhiboro AA, Tikhonova EP (Eds) War’s Tragic Memory: The Great Patriotic War in the Memoirs of the Staff of the Zoological Institute of the Russian Academy of Sciences. St. Petersburg: Russkaia Kollektsiya, pp. 322–325. [In Russian]

Petrova NA, Michailova PV (2021) The use of salivary gland chromosomes of chironomids (Diptera, Chironomidae) for assessing the pollution of aquatic ecosystems. Euroasian Entomological Journal 20(1): 38–48. [In Russian with English summary] <https://doi.org/10.15298/euroasentj.20.1.06>

2022

Petrova NA, Zhirov SV (2022) Structure of polytene chromosomes and larval morphology of chironomids (Diptera, Chironomidae). Atlas. St. Petersburg, Moscow: KMK Scientific Press, 114 pp., 155 plates. [In Russian]

2024

Petrova NA (2024) B-chromosomes of Chironomidae и Simuliidae (Diptera): a brief review. Euroasian Entomological Journal 23(4): 209–214. [In Russian with English summary] https://doi.org/10.15298/euroasentj.23.04.04
